# Supplementary material for: Induced fit with replica exchange improves protein complex structure prediction
Source: PLoS Comput Biol. 2022 Jun 3;18(6):e1010124. doi: 10.1371/journal.pcbi.1010124 (PMC9200320; doi:10.1371/journal.pcbi.1010124)
Supplement: S1 Table — (1) Nearest-native structures from rigid-body docking selected for refinement. (2) Half successes awarded for targets with multiple binding sites evaluated, where at least one but not all binding sites are captured. (3) 2.5 Å cutoff for near-native structures. (4) Cases where bootstrapping gives ≥50% chance of N5≥3 are considered successfully docked. (5) For CAPRI sets, medium and difficult targets are combined, comprising all targets without at least one high-quality prediction by any predictor. (6) Lensink et al. [14] (7) Hwang et al. [13] (8) Vreven et al. [12]. The ReplicaDock 2.0 and RosettaDock 4.0 test sets differ slightly because we omitted some easy targets and we added flexible targets that had been too large for the prior ensemble methods. (PDF) [file pcbi.1010124.s002.pdf]

Supplementary Tables

Table S1. Comparison of leading docking methods with ReplicaDock 2.0 (derived from Marze *et al.*(15)). (1) Nearest-native structures from rigid-body docking selected for refinement. (2) Half successes awarded for targets with multiple binding sites evaluated, where at least one but not all binding sites are captured. (3) 2.5 Å cutoff for near-native structures. (4) Cases where bootstrapping gives $\geq$50% chance of N5$\geq$3 are considered successfully docked. (5) For CAPRI sets, medium and difficult targets are combined, comprising all targets without at least one high-quality prediction by any predictor. (6) Lensink *et al.*(14) (7) Hwang *et al.*(13) (8) Vreven *et al.*(12). **The ReplicaDock 2.0 and RosettaDock 4.0 test sets differ slightly because we omitted some easy targets and we added flexible targets that had been too large for the prior ensemble methods.**

**Methods**						**Performance**		
Method	Description	Flexibility?	Benchmark Set	Docking Search	Success Metric	Easy Targets	Medium Targets[5]	Difficult Targets[5]
HADDOCK (2017)	Restraint-based docking, minimization	Yes	CASP-CAPRI[6]	Mixed global/local	N10 = 1	12/12 (100%)	4/13 (31%)	
ClusPro (2017)	FFT docking, cluster evaluation	No	CAPRI Rds. 13−35	Mixed global/local	N10 = 1	12.5[2]/16 (78%)	6.5[2]/26 (25%)	
iATTRACT (2015)	Rigid-body docking, interface refinement	Yes	Docking Benchmark 4.0[7]	Global[1]	N200 = 30	55/119 (46%)	9/28 (32%)	0/19 (0%)
ZDOCK (2011)	FFT docking, model evaluation	No	Docking Benchmark 4.0[7]	Global	N100 = 1[3]	58/121 (48%)	7/30 (23%)	0/25 (0%)
Rosetta Dock 3.2 (2011)	Monte Carlo docking, model evaluation	Yes	Docking Benchmark 4.0[7]	Local	N5 = 3	49/84 (58%)	5/17 (29%)	2/14 (14%)
RosettaDock 4.0 (2018)	Monte Carlo docking, model evaluation	Yes	Docking Benchmark 5.0[8]	Local	N5 = 3[4]	10/13 (77%)	21/43 (49%)	10/32 (31%)
ReplicaDock 2.0 (2021)	Replica Exchange Monte Carlo docking	Yes	Docking Benchmark 5.0[8]	Local	N5 > 3[4]	8/10 (80%)	27/44 (61%)	12/34 (35%)
