# Supplementary material for: Induced fit with replica exchange improves protein complex structure prediction
Source: PLoS Comput Biol. 2022 Jun 3;18(6):e1010124. doi: 10.1371/journal.pcbi.1010124 (PMC9200320; doi:10.1371/journal.pcbi.1010124)
Supplement: S2 Table — 5,000 decoys were generated by each protocol for each target. Bootstrapped N5 values (plus standard deviations), both after the low-resolution phase and after the full protocol, are listed for each target. Success is defined as 〈N5〉≥ 3 for the N5 metrics. (PDF) [file pcbi.1010124.s003.pdf]

Table S2. Performance of RosettaDock 4.0 vs. ReplicaDock 2.0 across an 88-target benchmark set. 5,000 decoys were generated by each protocol for each target. Bootstrapped N5 values (plus standard deviations), both after the low-resolution phase and after the full protocol, are listed for each target. Success is defined as ⟨N5⟩≥ 3 for the N5 metrics

Target	i-RMSD$_{u-b}$ (Å)	Difficulty	RosettaDock (LowRes)			RosettaDock			ReplicaDock (LowRes)			ReplicaDock		
1AY7	0.54	Rigid	0.6	±	0.8	5	±	0	5	±	0	5	±	0
1BVK	1.24	Rigid	0	±	0	4.9	±	0.5	3.2	±	1.1	3.6	±	1.1
1KTZ	0.39	Rigid	4.3	±	0.9	4.7	±	0.6	5	±	0	5	±	0
1MAH	0.61	Rigid	1.5	±	1.2	4.7	±	0.6	5	±	0.2	5	±	0
1MLC	0.6	Rigid	0	±	0.2	0.1	±	0.3	0	±	0	0	±	0
2BTF	0.75	Rigid	5	±	0.3	5	±	0	5	±	0	0.6	±	0.9
2JEL	0.17	Rigid	3.7	±	1.3	4.5	±	0.8	5	±	0	5	±	0
2PCC	0.39	Rigid	0	±	0	3	±	1.4	5	±	0	5	±	0
2SIC	0.36	Rigid	0.6	±	0.8	5	±	0	5	±	0	5	±	0
2SNI	0.35	Rigid	4.1	±	0.9	5	±	0	5	±	0	5	±	0
1B6C	1.96	Medium	5	±	0	5	±	0	5	±	0.1	5	±	0
1CGI	2.02	Medium	1.9	±	1.3	1.9	±	1.4	5	±	0	3	±	1.1
1FC2	1.69	Medium	0	±	0.2	0	±	0.2	0.2	±	0.5	3.1	±	1.3
1GP2	1.65	Medium	1.6	±	1.1	1.6	±	1.1	0	±	0	0	±	0
1GRN	1.22	Medium	1.4	±	1.1	1.3	±	1	5	±	0	5	±	0
1HE8	0.92	Medium	4.5	±	0.7	4.6	±	0.7	5	±	0	5	±	0
1I2M	2.12	Medium	0	±	0	0	±	0	5	±	0	4.6	±	0.8
1IB1	2.09	Medium	0	±	0	0	±	0	0	±	0	0	±	0
1IJK	0.68	Medium	3.7	±	1.1	3.7	±	1	5	±	0	5	±	0
1JIW	2.07	Medium	0.8	±	1.1	0.9	±	1.1	0.1	±	0.3	2.5	±	1.2
1K5D	1.19	Medium	0.9	±	0.9	0.9	±	0.9	0	±	0.1	1.2	±	1
1KKL	2.2	Medium	3.1	±	1.2	3.2	±	1.2	5	±	0	0	±	0
1LFD	1.79	Medium	5	±	0	5	±	0	5	±	0	5	±	0
1M10	2.1	Medium	0	±	0.2	0	±	0.2	0.2	±	0.5	3	±	1.2
1MQ8	1.76	Medium	5	±	0	5	±	0	0	±	0	2.3	±	1.3
1N2C	2.13	Medium	1.3	±	0.8	0	±	0	5	±	0	0.1	±	0.3
1NW9	1.97	Medium	3.6	±	1.1	3.6	±	1.1	0	±	0	1.4	±	1.1
1R6Q	1.67	Medium	2.7	±	1.3	2.7	±	1.3	5	±	0	5	±	0
1SYX	1.64	Medium	5	±	0	5	±	0	5	±	0	5	±	0
1WQ1	1.16	Medium	2.8	±	1.3	2.8	±	1.3	5	±	0	4.6	±	0.6
1XQS	1.77	Medium	4.2	±	0.9	4.2	±	0.8	5	±	0	4.3	±	0.8
1ZM4	2.11	Medium	2.5	±	1.1	2.5	±	1.2	0.5	±	0.9	4.9	±	0.3
2CFH	1.55	Medium	5	±	0	5	±	0	5	±	0	5	±	0
2H7V	1.63	Medium	2.6	±	1.1	2.6	±	1.2	5	±	0	3	±	1.1
2HRK	2.03	Medium	4.4	±	0.8	4.4	±	0.9	1.2	±	1	4.9	±	0.4
2NZ8	2.13	Medium	3.6	±	1.1	3.7	±	1.1	3.2	±	1.1	3.6	±	1.1

Target	i-RMSD$_{u-b}$ (Å)	Difficulty	RosettaDock (LowRes)			RosettaDock			ReplicaDock (LowRes)			ReplicaDock		
2OZA	1.89	Medium	0	±	0	0	±	0	0	±	0	0	±	0
2Z0E	2.15	Medium	0.9	±	0.9	0.9	±	0.9	0.1	±	0.5	0.1	±	0.4
3AAA	1.78	Medium	1.5	±	1.1	1.5	±	1.1	0	±	0.2	0	±	0
3AAD	2	Medium	0.9	±	0.9	0.9	±	0.9	0	±	0	0	±	0
3BX7	1.63	Medium	2.4	±	1.4	2.5	±	1.3	5	±	0	2.6	±	1.2
3CPH	2.12	Medium	0.4	±	0.7	0.4	±	0.7	0.7	±	0.9	2.1	±	1.2
3DAW	1.49	Medium	4.4	±	0.8	4.4	±	0.8	5	±	0	3.1	±	1.1
3EO1	1.37	Medium	5	±	0.2	5	±	0.2	5	±	0	5	±	0
3G6D	1.86	Medium	1.1	±	1	1.1	±	1.1	0	±	0.1	5	±	0
3HI6	1.65	Medium	0	±	0.1	0	±	0.2	0	±	0	0.4	±	0.7
3L5W	0.48	Medium	5	±	0	5	±	0	5	±	0	3.8	±	1
3S9D	1.69	Medium	0.8	±	0.9	3.9	±	1.1	0.1	±	0	4.5	±	0.8
3SZK	2.1	Medium	3.4	±	1.2	3.3	±	1.2	5	±	0	5	±	0
3V6Z	1.83	Medium	0	±	0	0	±	0	5	±	0	2.1	±	1.2
4FZA	2.04	Medium	1	±	1	1	±	1	1.6	±	1.2	5	±	0
4IZ7	1.56	Medium	0	±	0.1	0	±	0.1	0	±	0	0	±	0
4JCV	1.62	Medium	2.5	±	1.1	2.6	±	1.2	2.6	±	1.3	5	±	0
4LW4	1.6	Medium	1.6	±	1.1	1.5	±	1.1	4.7	±	0.8	3.7	±	1.3
1ACB	2.26	Difficult	2.3	±	1.2	2.3	±	1.2	5	±	0.1	3.2	±	1.2
1ATN	3.28	Difficult	1.9	±	1.1	1.9	±	1.1	4.7	±	0.6	2.5	±	1.2
1BGX	6.91	Difficult	0	±	0	0	±	0	0	±	0	0	±	0
1BKD	2.86	Difficult	0	±	0	0	±	0	0	±	0	0	±	0
1DE4	2.59	Difficult	2.3	±	1.2	2.3	±	1.2	0	±	0	4.8	±	0.5
1E4K	2.6	Difficult	0	±	0.2	0	±	0.2	0	±	0	0	±	0
1EER	2.44	Difficult	0.1	±	0.4	0.1	±	0.4	5	±	0.2	0	±	0
1F6M	4.9	Difficult	0.3	±	0.7	0.2	±	0.6	0	±	0	0	±	0
1FAK	6.18	Difficult	0	±	0.2	0	±	0.2	0	±	0	0	±	0
1FQ1	3.41	Difficult	5	±	0.1	5	±	0.1	4.9	±	0.4	5	±	0
1H1V	6.62	Difficult	0	±	0	0	±	0	0	±	0	0	±	0
1IBR	2.54	Difficult	0	±	0	0	±	0	0	±	0	0	±	0
1IRA	8.38	Difficult	0	±	0	0	±	0	0	±	0	0	±	0
1JK9	2.51	Difficult	5	±	0	5	±	0	5	±	0	5	±	0
1JMO	3.21	Difficult	1.4	±	1	1.4	±	1	4.6	±	0.8	1.6	±	1.2
1JZD	2.71	Difficult	1.9	±	1.3	1.9	±	1.2	5	±	0	5	±	0
1PXV	2.63	Difficult	2.6	±	1.3	2.5	±	1.3	1.3	±	1.1	3.2	±	0.6
1R8S	3.73	Difficult	0	±	0	0	±	0	0.1	±	0.3	0	±	0.1

Target	i-RMSD$_{u-b}$ (Å)	Difficulty	RosettaDock (LowRes)			RosettaDock			ReplicaDock (LowRes)			ReplicaDock		
1RKE	4.25	Difficult	0	±	0.1	0	±	0.1	0	±	0	1.6	±	1.2
1Y64	4.69	Difficult	0	±	0	0	±	0	0	±	0	0	±	0
1ZLI	2.53	Difficult	0.8	±	0.9	0.7	±	0.8	1.2	±	1	0	±	0
2C0L	2.62	Difficult	4	±	1	4	±	1	0	±	0	0	±	0
2HMI	2.26	Difficult	2.9	±	1.3	2.9	±	1.3	0	±	0	0	±	0.1
2I9B	3.79	Difficult	1	±	1	1.1	±	1	0.1	±	0.4	2.2	±	1.2
2IDO	2.79	Difficult	3.7	±	1.1	3.7	±	1	5	±	0	4	±	0.9
2J7P	2.67	Difficult	0	±	0	0	±	0	0	±	0	0	±	0
2O3B	3.13	Difficult	1.4	±	1.1	1.3	±	1.1	1.4	±	1.4	4	±	1
2OT3	2.79	Difficult	0	±	0	0	±	0	5	±	0	0	±	0
3AAD	4.37	Difficult	0	±	0	0	±	0	0	±	0	0	±	0
3F1P	2.52	Difficult	5	±	0.1	5	±	0.1	5	±	0	5	±	0
3FN1	3.65	Difficult	1.5	±	1.2	1.5	±	1.1	5	±	0	4.9	±	0.5
3H11	3.79	Difficult	5	±	0	5	±	0	5	±	0	5	±	0
3L89	2.51	Difficult	4.3	±	0.8	4.4	±	0.8	5	±	0	5	±	0
4GAM	5.79	Difficult	2.3	±	1.2	2.4	±	1.2	0	±	0	0	±	0
