# Supplementary material for: Induced fit with replica exchange improves protein complex structure prediction
Source: PLoS Comput Biol. 2022 Jun 3;18(6):e1010124. doi: 10.1371/journal.pcbi.1010124 (PMC9200320; doi:10.1371/journal.pcbi.1010124)
Supplement: S1 Fig — (PDF) [file pcbi.1010124.s004.pdf]

Supplementary Figures

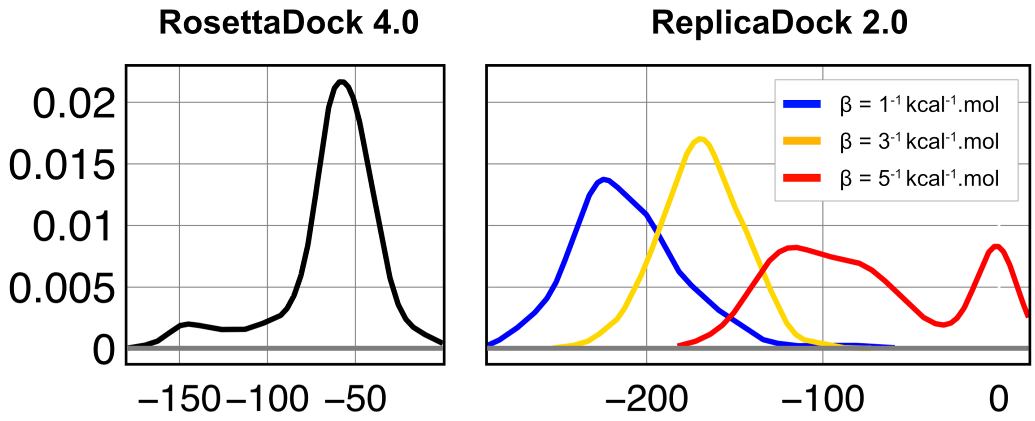

Fig. S1. Energy distribution of conformations sampled with RosettaDock 4.0 and ReplicaDock 2.0 (at respective inverse temperatures) for protein target 2CFH
