# Supplementary material for: Induced fit with replica exchange improves protein complex structure prediction
Source: PLoS Comput Biol. 2022 Jun 3;18(6):e1010124. doi: 10.1371/journal.pcbi.1010124 (PMC9200320; doi:10.1371/journal.pcbi.1010124)
Supplement: S2 Fig — Interface score (REU) vs I-rmsd(Å) for each of the 10 benchmark targets, arranged by target difficulty. ReplicaDock 2.0 decoys colored in gray and ClusPro models, relaxed with Rosetta and scored with MUDS, highlighted in red. (PDF) [file pcbi.1010124.s005.pdf]

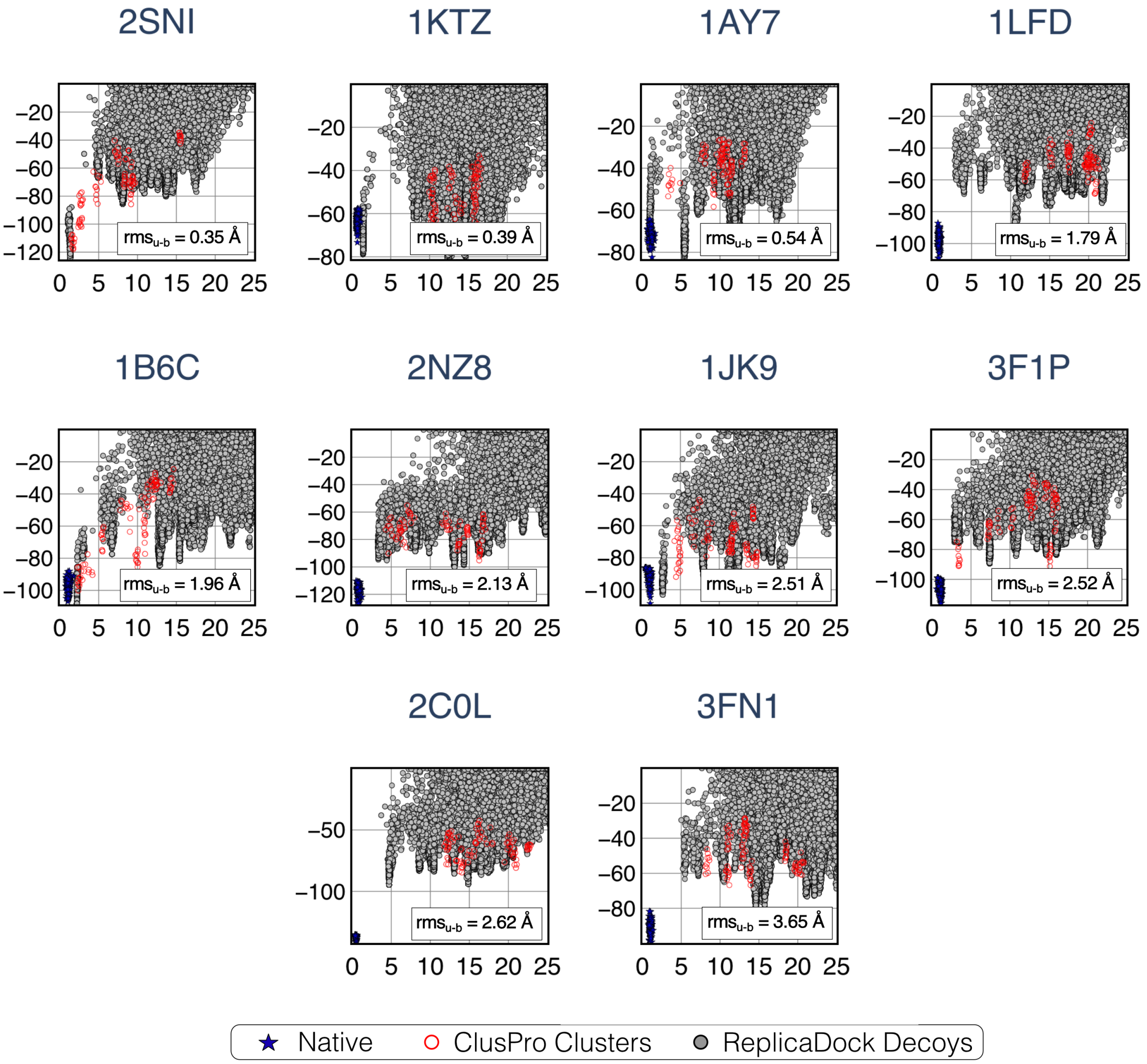

Fig. S2. Global docking performance. Interface score (REU) vs I-rmsd (Å) for each of the 10 benchmark targets, arranged by target difficulty. ReplicaDock 2.0 decoys colored in gray and ClusPro models, relaxed with Rosetta and scored with MUDS, highlighted in red.
