# Supplementary material for: Induced fit with replica exchange improves protein complex structure prediction
Source: PLoS Comput Biol. 2022 Jun 3;18(6):e1010124. doi: 10.1371/journal.pcbi.1010124 (PMC9200320; doi:10.1371/journal.pcbi.1010124)
Supplement: S3 Fig — (A) ReplicaDock2.0 prediction (green-blue) superimposed over the bound structure (in gray-tan). Comparison of interface regions between for bound (gray-tan) with ReplicaDock2.0 model (green-blue) and AlphaFold (pale green-pale blue) highlighted below. AlphaFold prediction (pale green-pale blue) superimposed over the bound structure. Binding orientation correlates with the wildtype, but prediction derives mostly from the unbound templates. AlphaFold-multimer was used via available jupyter notebook, but the models generated with AlphaFold-multimer were almost identical (less than 0.5 Å all-atom RMSD difference) to the models produced with AlphaFold. (B) Top-5 predictions for the predictor groups colored by the prediction quality. AlphaFold and ReplicaDock2 capture better quality decoys than predictions. RoseTTAFold predictions are inaccurate. (C) Interface-RMSD (Å) for the top-5 predictions by the predictor groups. RoseTTAFold models could not capture models within 8 Å and are excluded from this plot. (PDF) [file pcbi.1010124.s006.pdf]

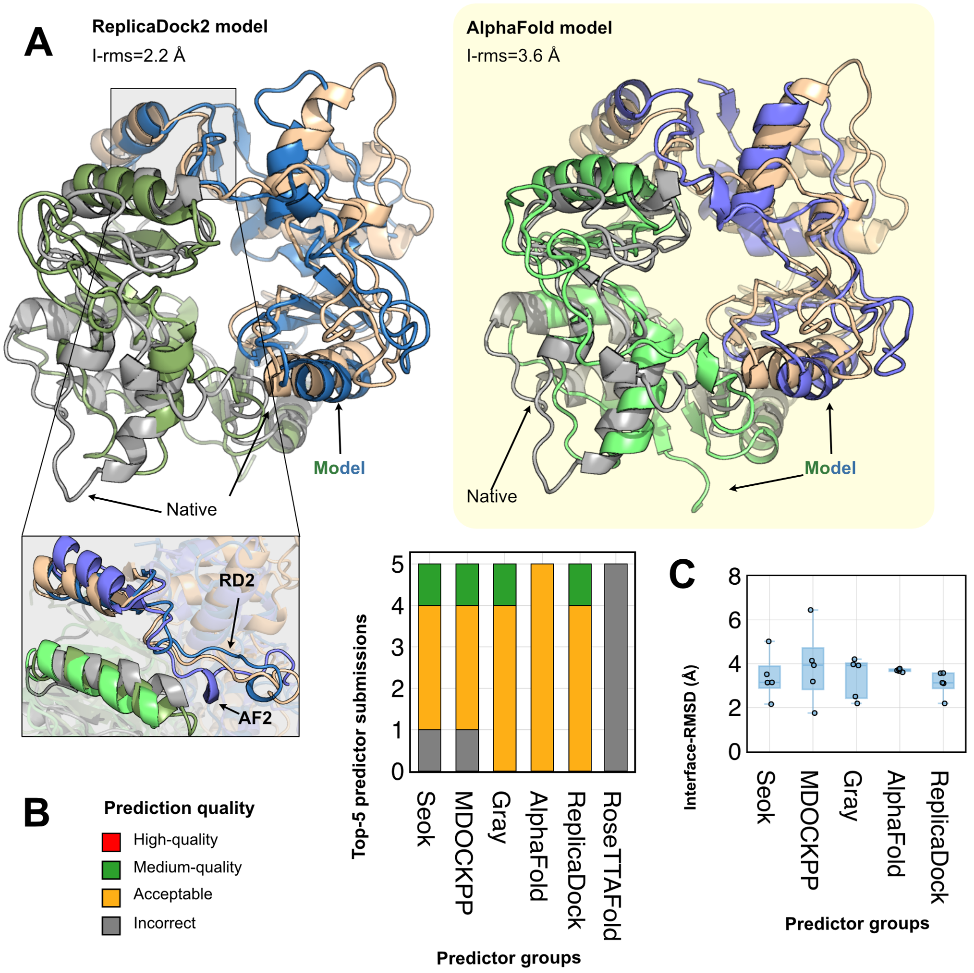

Fig. S3. ReplicaDock2 and AlphaFold performances on blind CAPRI target T164. (A) ReplicaDock2.0 prediction (green-blue) superimposed over the bound structure (in gray-tan). Comparison of interface regions between for bound (gray-tan) with ReplicaDock2.0 model (green-blue) and AlphaFold (pale green-pale blue) highlighted below. AlphaFold prediction (pale green-pale blue) superimposed over the bound structure. Binding orientation correlates with the wildtype, but prediction derives mostly from the unbound templates. AlphaFold-multimer was used via available jupyter notebook, but the models generated with AlphaFold-multimer were almost identical (less than 0.5 Å all-atom RMSD difference) to the models produced with AlphaFold. (B) Top-5 predictions for the predictor groups colored by the prediction quality. AlphaFold and ReplicaDock2 capture better quality decoys than predictions. RoseTTAFold predictions are inaccurate. (C) Interface-RMSD (Å) for the top-5 predictions by the predictor groups. RoseTTAFold models could not capture models within 8 Å and are excluded from this plot.
