# Supplementary material for: Induced fit with replica exchange improves protein complex structure prediction
Source: PLoS Comput Biol. 2022 Jun 3;18(6):e1010124. doi: 10.1371/journal.pcbi.1010124 (PMC9200320; doi:10.1371/journal.pcbi.1010124)
Supplement: S4 Fig — The four residues selections are as follows: (1) 5.5 Å interface patch, (2) 8 Å interface patch (3) 5.5 Å interface patch + loops, (4) 8 Å interface patch + loops. Note that, we also performed a test set by including all the residues of the protein for backbone sampling, however, with T-REMC, such simulations resulted in distortion of the protein quaternary structure (i.e. resulted in protein unfolding). Therefore, we chose to exclude that test. (PDF) [file pcbi.1010124.s007.pdf]

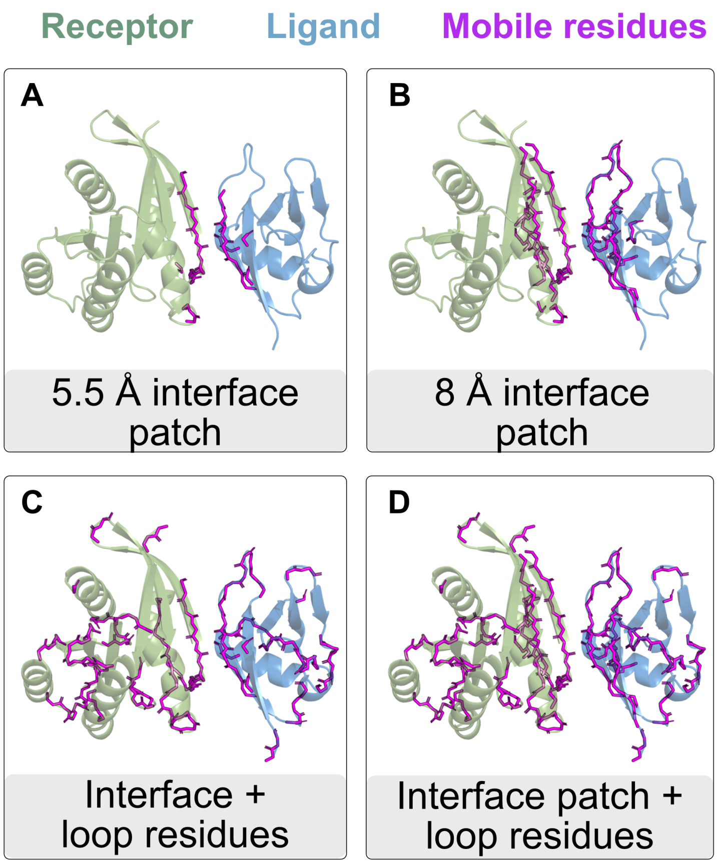

Benchmarking targets

Difficult targets	Medium targets	Rigid targets
1FQ1	1GRN	1AY7
1JK9	1IJK	1MAH
3F1P	3DAW	2PCC
3FN1	4FZA	2SNI

Fig. S4. Interface residue selections (*in magenta*) highlighted over a protein target (Receptor, *in green* and ligand, *in blue*). The four residues selections are as follows: (1) 5.5 Å interface patch, (2) 8 Å interface patch (3) 5.5 Å interface patch + loops, (4) 8 Å interface patch + loops. Note that, we also performed a test set by including all the residues of the protein for backbone sampling, however, with T-REMC, such simulations resulted in distortion of the protein quaternary structure (i.e. resulted in protein unfolding). Therefore, we chose to exclude that test.
