# Supplementary material for: Induced fit with replica exchange improves protein complex structure prediction
Source: PLoS Comput Biol. 2022 Jun 3;18(6):e1010124. doi: 10.1371/journal.pcbi.1010124 (PMC9200320; doi:10.1371/journal.pcbi.1010124)
Supplement: S5 Fig — Mobile residues sets are as follows: (left) 8 Å interface patch, and (right) 8 Å interface patch + loops. Candidate structures are colored by the CAPRI quality post all-atom refinement (colors: green = high quality, red = moderate quality, yellow = acceptable quality, gray = incorrect). (PDF) [file pcbi.1010124.s008.pdf]

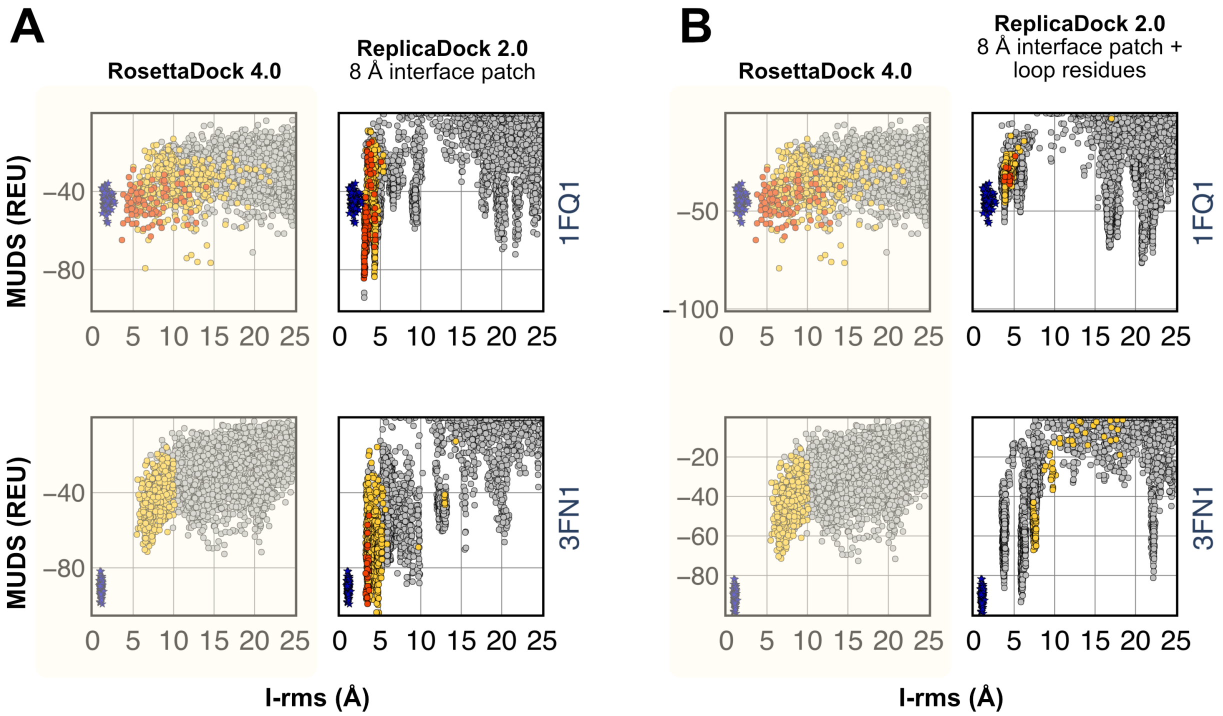

Fig. S5. MUDS versus Cα-RMSD(Å) plots for RosettaDock 4.0 and ReplicaDock 2.0 for two sets of residue selections in the low-resolution stage. Mobile residues sets are as follows: (left) 8 Å interface patch, and (right) 8 Å interface patch + loops. Candidate structures are colored by the CAPRI quality post all-atom refinement (colors : green = high quality, red = moderate quality, yellow = acceptable quality, gray = incorrect).
