# Supplementary material for: Induced fit with replica exchange improves protein complex structure prediction
Source: PLoS Comput Biol. 2022 Jun 3;18(6):e1010124. doi: 10.1371/journal.pcbi.1010124 (PMC9200320; doi:10.1371/journal.pcbi.1010124)
Supplement: S6 Fig — A. Near-native enrichment for two different weights based on MUDS. Each score-term is represented with the boxplot illustrating the enrichment of CAPRI-quality accceptable models within the lowest-scoring 1,000 models out of a set of 10,000 (10%) for each of the 11 protein-protein complexes. The motif-dock score corresponds to the score function defined in Marze et al. [15]. Motif Updated Dock Score(MUDS) in blue (as defined prior) comprises of Van der Waals score terms(attractive and repulsive clash terms) in pale orange and Backbone statistical terms(rama, p_aa_pp and omega) in pale green along with the motif-dock score in purple. (left) Represents reduced weights of 0.1X to the Van der Waals and the backbone score terms, where X is the standard weight as defined by beta_nov16. (right) Represents a weight of 1.0X to the score-terms where X is the standard weight defined by the beta_nov16 score-function. Sampling performance is improved almost three-fold for all 11 targets by improved weights. B. Distribution of protein targets to compare scoring performance. (PDF) [file pcbi.1010124.s009.pdf]

A

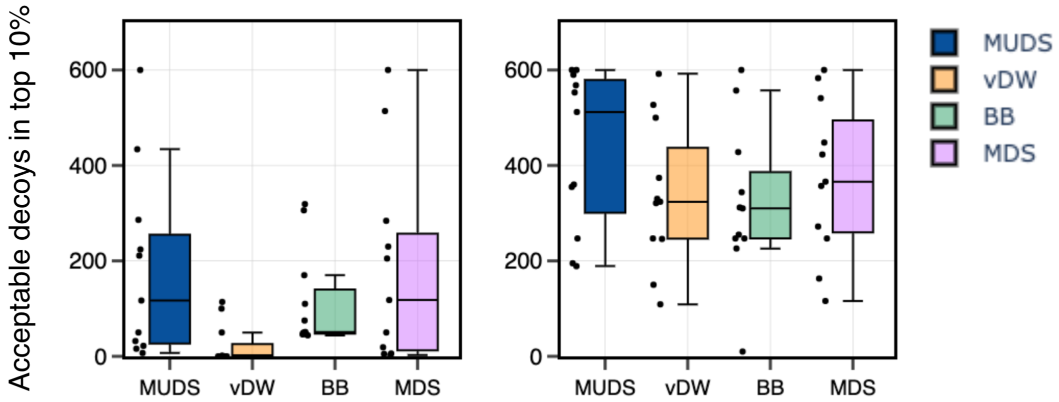

B

Difficult targets	Medium targets	Rigid targets
1FQ1<br>3FN1<br>3H11	1GRN<br>1IJK<br>1LFD<br>3DAW<br>4FZA	1AY7<br>1MAH<br>2PCC<br>2SNI

Fig. S6. A. **Near-native enrichment** for two different weights based on MUDS. Each score-term is represented with the boxplot illustrating the enrichment of CAPRI-quality accceptable models within the lowest-scoring 1,000 models out of a set of 10,000 (10%) for each of the 11 protein-protein complexes. The motif-dock score corresponds to the score function defined in *Marze et al.* (13). Motif Updated Dock Score(MUDS) *in blue* (as defined prior) comprises of Van der Waals score terms(attractive and repulsive clash terms) *in pale orange* and Backbone statistical terms(`rama`, `p_aa_pp` and `omega`) *in pale green* along with the motif-dock score *in purple*. (left) Represents reduced weights of 0.1X to the Van der Waals and the backbone score terms, where X is the standard weight as defined by `beta_nov16`. (right) Represents a weight of 1.0X to the score-terms where X is the standard weight defined by the `beta_nov16` score-function. Sampling performance is improved almost three-fold for all 11 targets by improved weights. B. Distribution of protein targets to compare scoring performance
