# Supplementary material for: Induced fit with replica exchange improves protein complex structure prediction
Source: PLoS Comput Biol. 2022 Jun 3;18(6):e1010124. doi: 10.1371/journal.pcbi.1010124 (PMC9200320; doi:10.1371/journal.pcbi.1010124)
Supplement: S7 Fig — (A) Native structure (PDB ID: 6N64) in grey, superimposed by the available complex template (PDB ID: 1GXK) in light-blue, with an interface RMS of 3.84 Å. (B) AlphaFold model (green-blue) superimposed over the complex template (in light-blue). AlphaFold models the protein complex closer to the complex template, with considerable conformational differences amounting to an interface RMSD of 2.16 Å. (C) AlphaFold model superimposed over the native shows an interface RMSD of 3.7 Å. Although marked as an easy target due to the availability of the template, this target comprises flexible loops and helical rearrangements. (PDF) [file pcbi.1010124.s010.pdf]

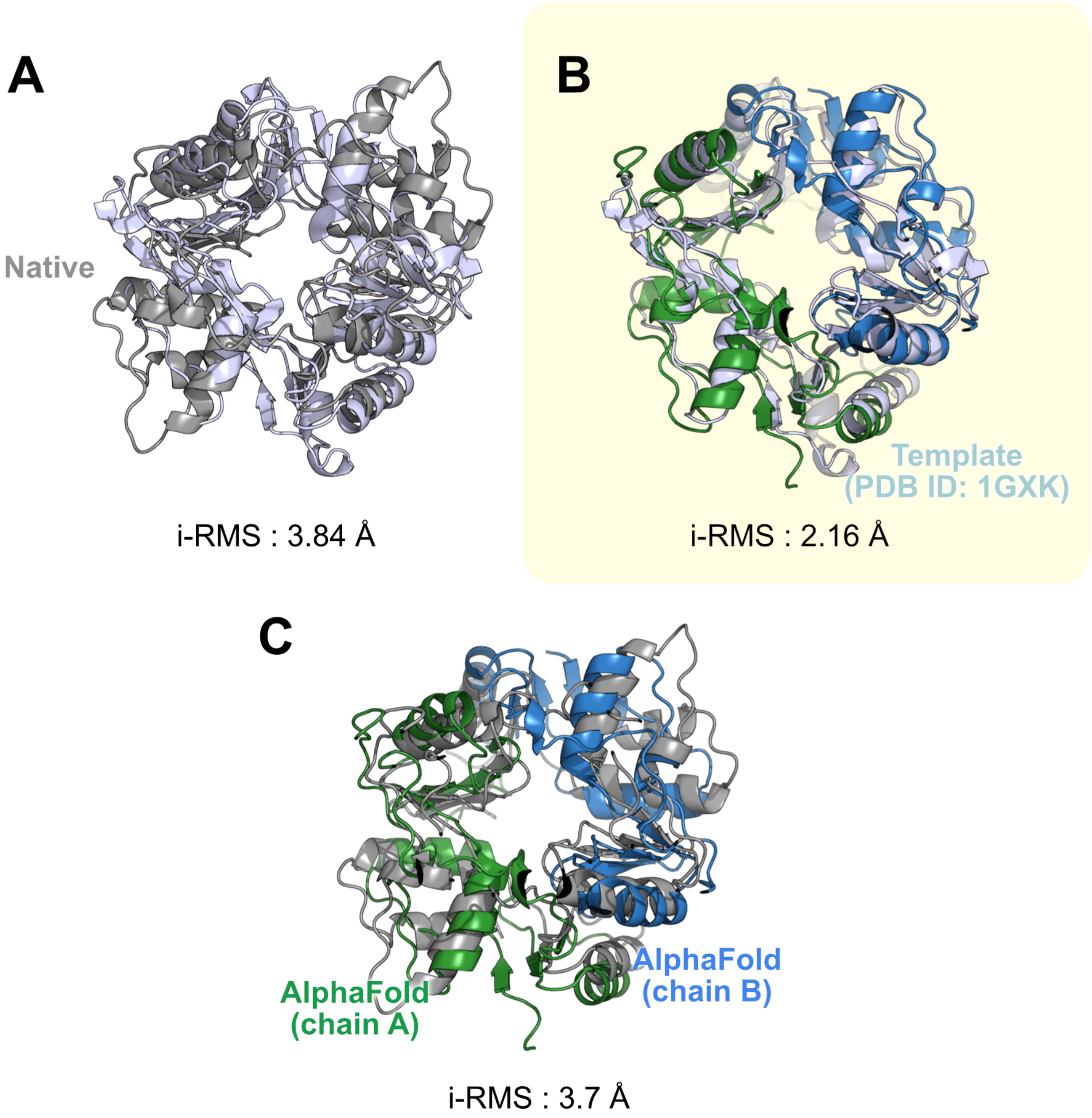

Fig. S7. AlphaFold complex modeling for CASP14-CAPRI target T164, comprising of the SMCHD1 (human) residues 1616-1899 Structural maintenance of chromosomes flexible hinge domain-containing protein 1. (A) Native structure (PDB ID: 6N64) in grey, superimposed by the available complex template (PDB ID: 1GXK) in light-blue, with an interface RMS of 3.84 Å. (B) AlphaFold model (green-blue) superimposed over the complex template (in light-blue). AlphaFold models the protein complex closer to the complex template, with considerable conformational differences amounting to an interface RMSD of 2.16 Å. (C) AlphaFold model superimposed over the native shows an interface RMSD of 3.7 Å. Although marked as an easy target due to the availability of the template, this target comprises flexible loops and helical rearrangements.
