# Supplementary material for: Induced fit with replica exchange improves protein complex structure prediction
Source: PLoS Comput Biol. 2022 Jun 3;18(6):e1010124. doi: 10.1371/journal.pcbi.1010124 (PMC9200320; doi:10.1371/journal.pcbi.1010124)
Supplement: S8 Fig — (A) Interface Score (REU) vs Interface-RMSD (Å) plots show that ReplicaDock captures acceptable decoys (and a few medium-quality decoys) starting from the complex template, however, if flexible docking is initiated with AlphaFold structures, we improve the quality of our predictions by 0.6–0.8 Å and capture more medium-quality decoys with better interface scores. (B) The models from ReplicaDock with (green-blue) and without(palegreen-paleblue) AlphaFold template are superimposed over the native(grey-tan). (PDF) [file pcbi.1010124.s011.pdf]

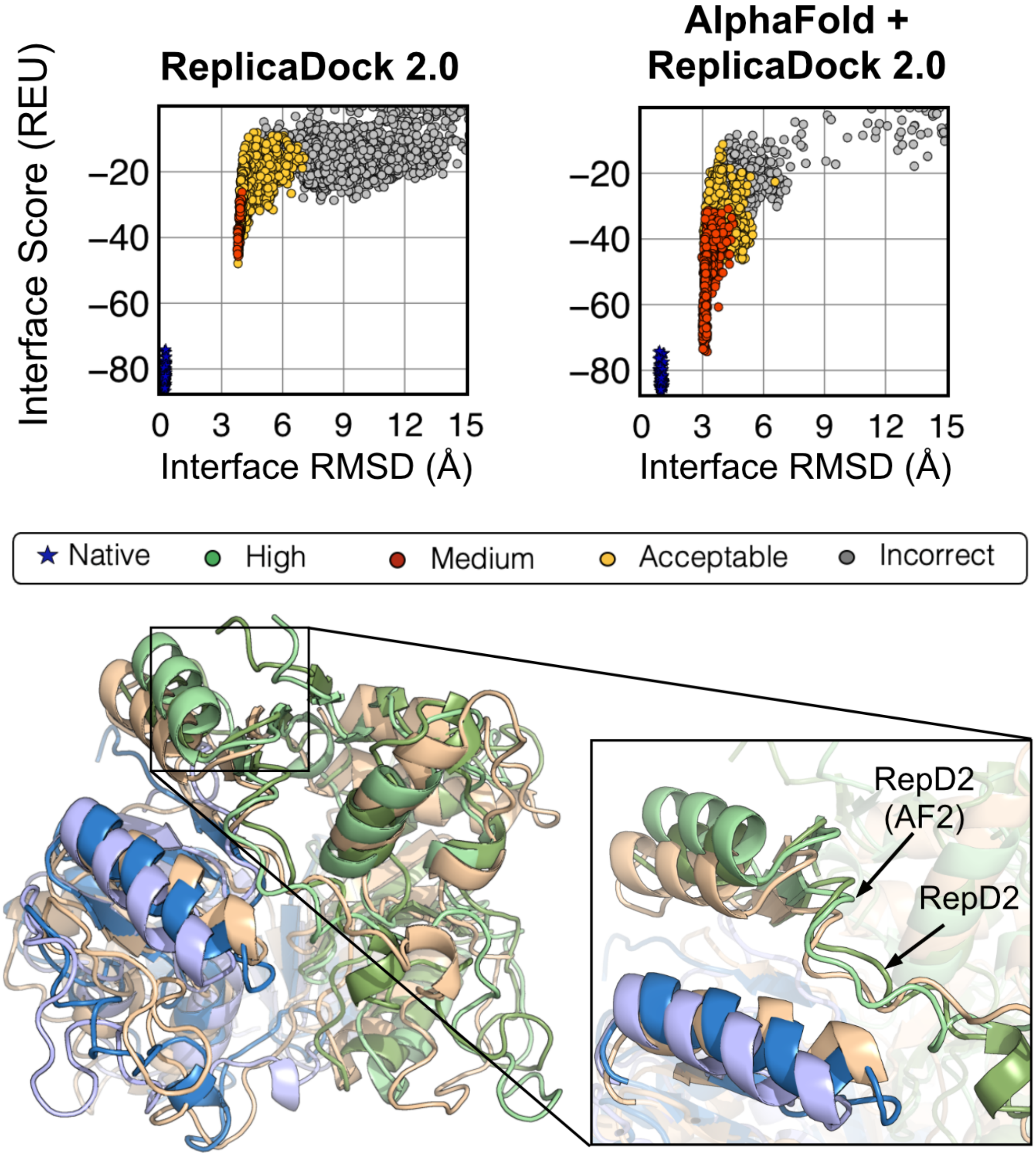

Fig. S8. ReplicaDock 2.0 performance on target T164 with and without AlphaFold template. (A) Interface Score (REU) vs Interface-RMSD (Å) plots show that ReplicaDock captures acceptable decoys (and a few medium-quality decoys) starting from the complex template, however, if flexible docking is initiated with AlphaFold structures, we improve the quality of our predictions by 0.6-0.8 Å and capture more medium-quality decoys with better interface scores. (B) The models from ReplicaDock with (green-blue) and without(palegreen-paleblue) AlphaFold template are superimposed over the native(grey-tan)
