# Supplementary material for: Induced fit with replica exchange improves protein complex structure prediction
Source: PLoS Comput Biol. 2022 Jun 3;18(6):e1010124. doi: 10.1371/journal.pcbi.1010124 (PMC9200320; doi:10.1371/journal.pcbi.1010124)
Supplement: S9 Fig — AlphaFold predictions for complex predictions involved broken tertiary structures and are not reported. ReplicaDock found acceptable quality targets, however, the H3 loop was not adequately sampled owing to the relatively lower Interface-RMSD. (PDF) [file pcbi.1010124.s012.pdf]

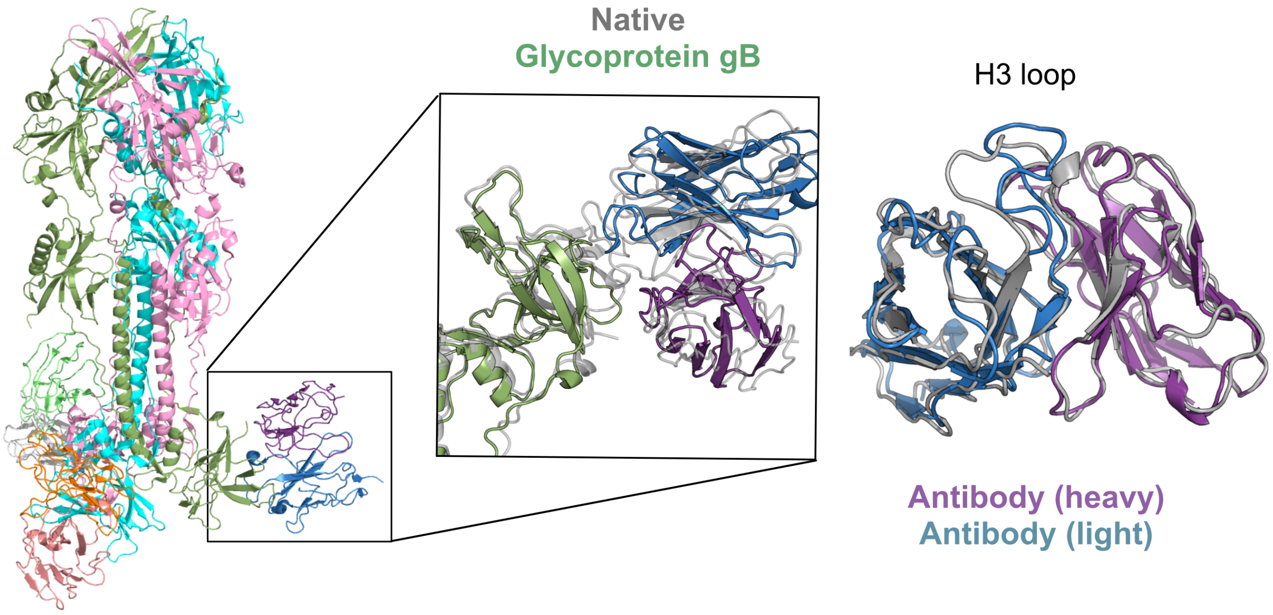

Fig. S9. ReplicaDock 2.0 performance on target T165 (a monoclonal antibody 93k bound to varicella-zoster virus glycoprotein gB). AlphaFold predictions for complex predictions involved broken tertiary structures and are not reported. ReplicaDock found acceptable quality targets, however, the H3 loop was not adequately sampled owing to the relatively lower Interface-RMSD.
