# Supplementary material for: Induced fit with replica exchange improves protein complex structure prediction
Source: PLoS Comput Biol. 2022 Jun 3;18(6):e1010124. doi: 10.1371/journal.pcbi.1010124 (PMC9200320; doi:10.1371/journal.pcbi.1010124)
Supplement: S11 Fig — (PDF) [file pcbi.1010124.s014.pdf]

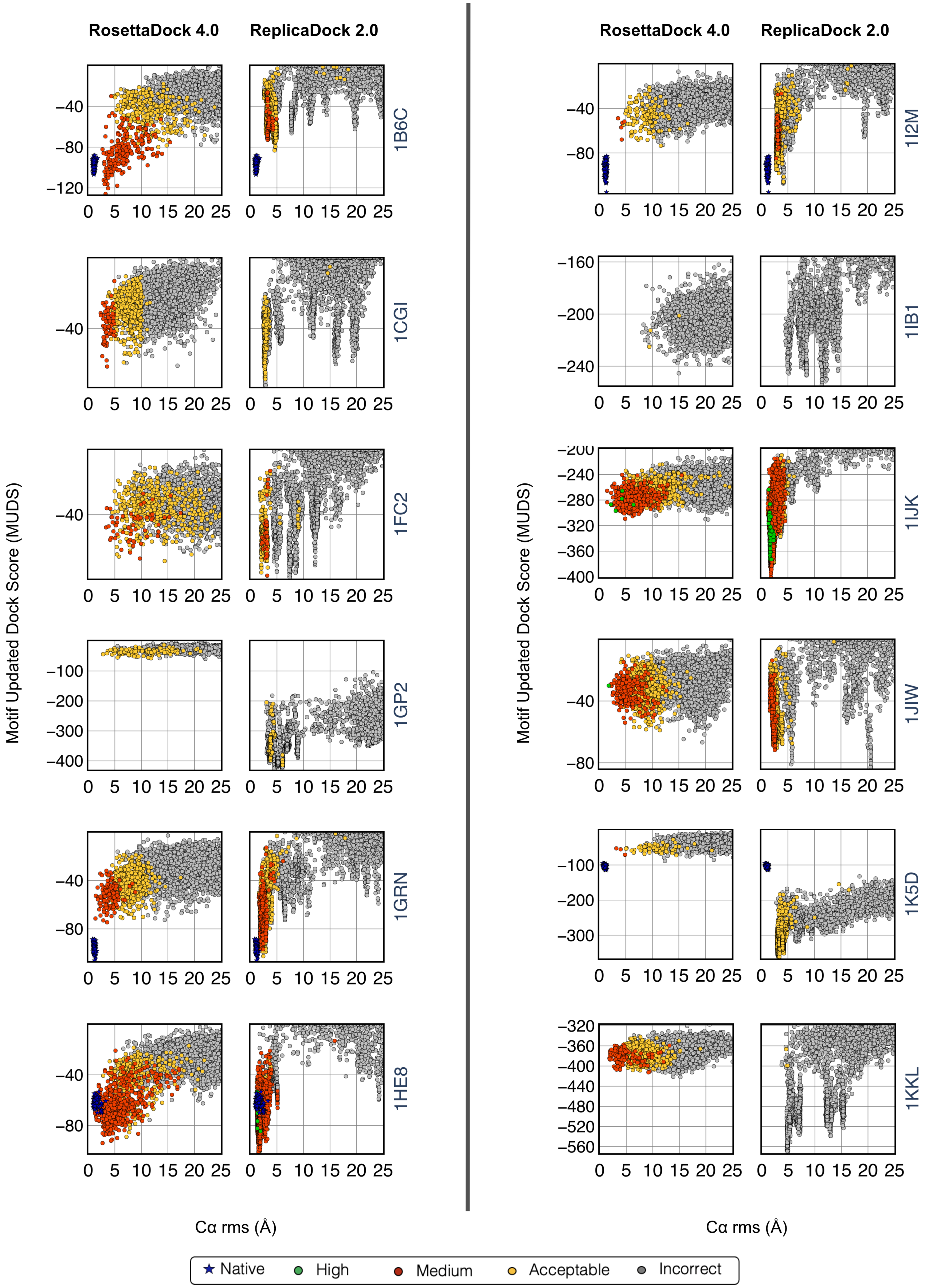

RosettaDock 4.0
ReplicaDock 2.0
RosettaDock 4.0
ReplicaDock 2.0
1B6C
1CGI
1FC2
1GP2
1GRN
1HE8
1I2M
1IB1
1IJK
1JIW
1K5D
1KKL
Motif Updated Dock Score (MUDS)
Cα rms (Å)
Native
High
Medium
Acceptable
Incorrect

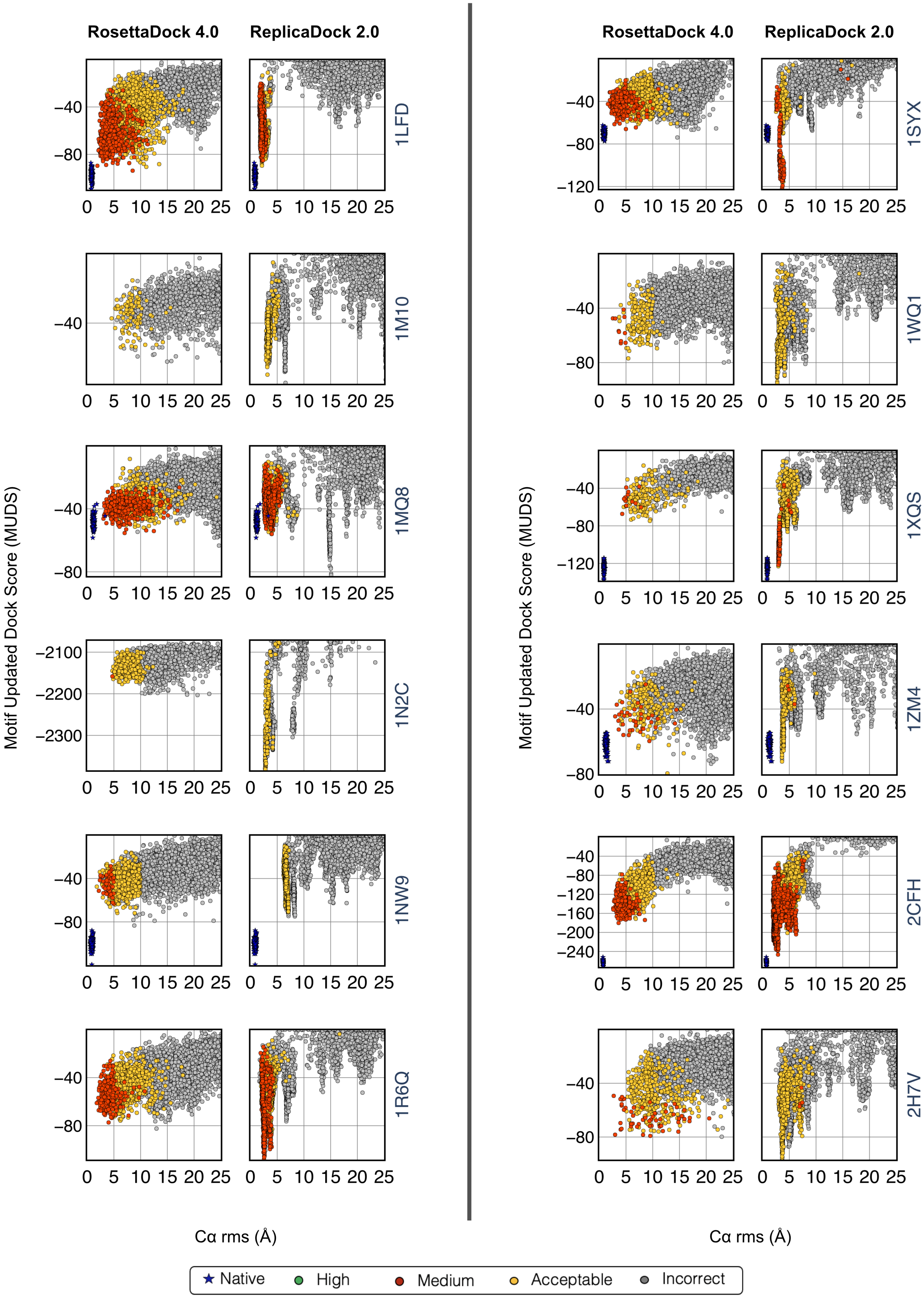
RosettaDock 4.0
ReplicaDock 2.0
RosettaDock 4.0
ReplicaDock 2.0
1LFD
1M10
1MQ8
1N2C
1NW9
1R6Q
1SYX
1WQ1
1XQS
1ZM4
2CFH
2H7V
Motif Updated Dock Score (MUDS)
Motif Updated Dock Score (MUDS)
Cα rms (Å)
Cα rms (Å)
Native
High
Medium
Acceptable
Incorrect

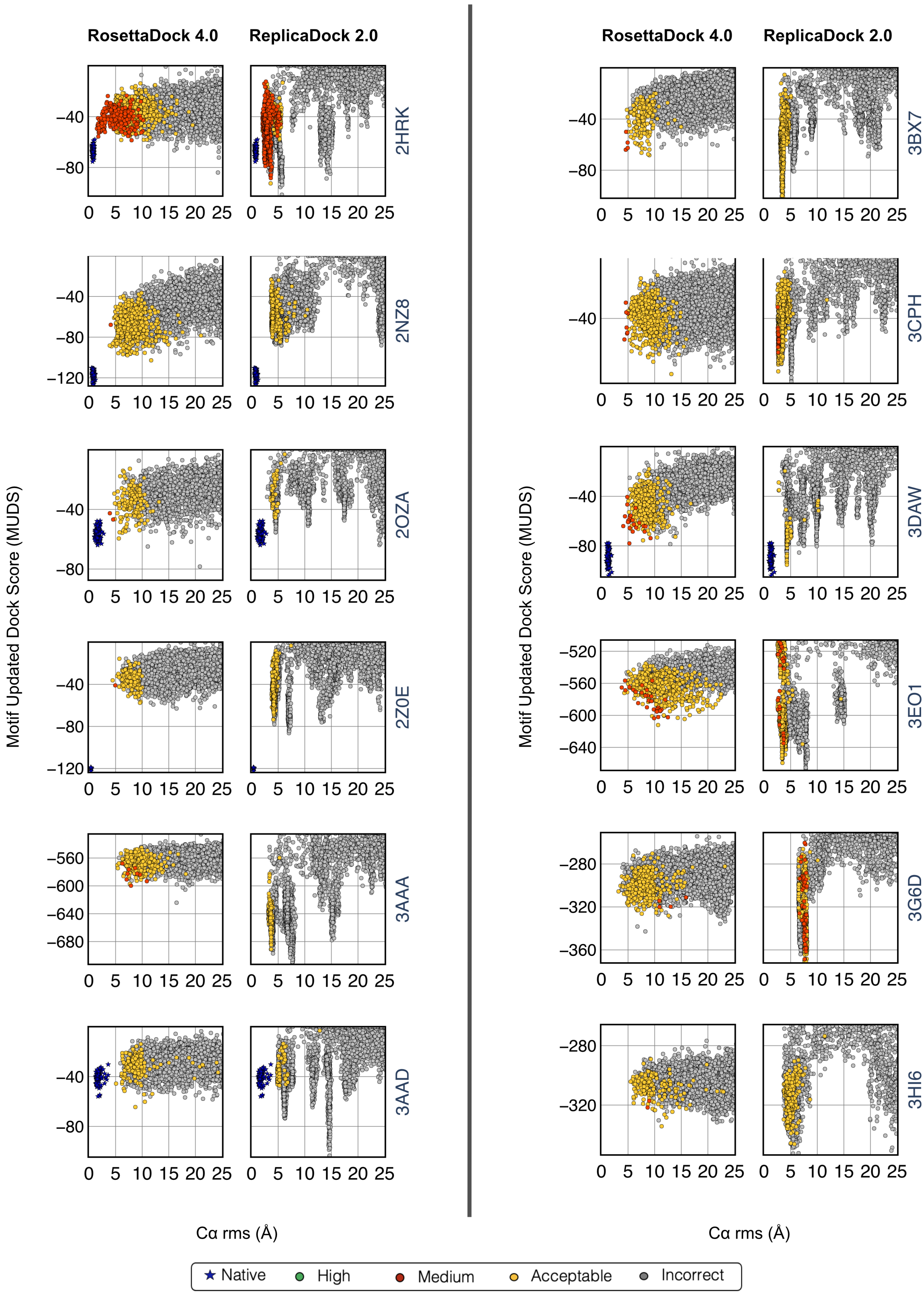
RosettaDock 4.0
ReplicaDock 2.0
RosettaDock 4.0
ReplicaDock 2.0
2HRK
2NZ8
2OZA
2Z0E
3AAA
3AAD
3BX7
3CPH
3DAW
3EO1
3G6D
3HI6
Motif Updated Dock Score (MUDS)
Cα rms (Å)
Native
High
Medium
Acceptable
Incorrect

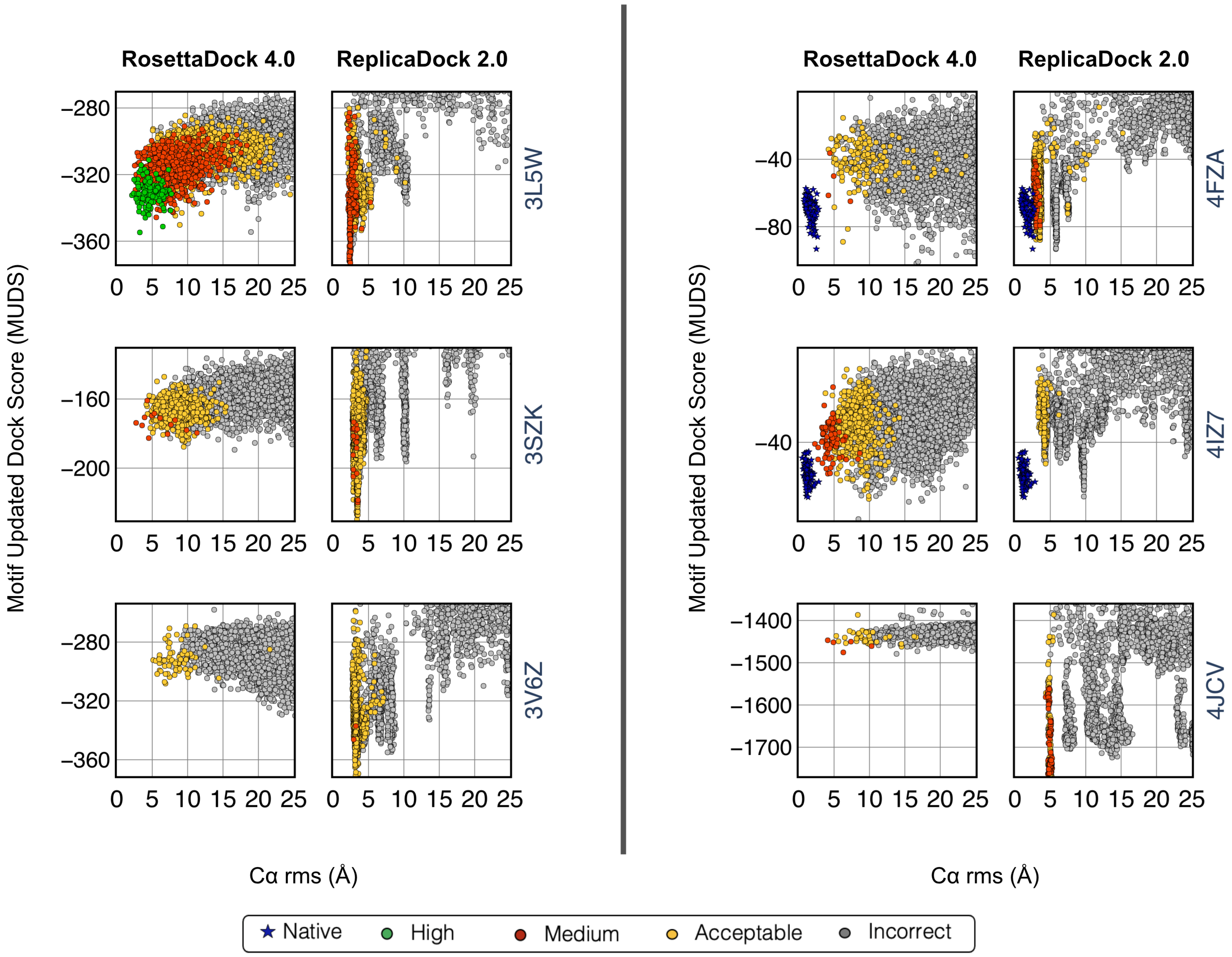

Fig. S11. Score versus Cα-RMSD(Å) plots in the low-resolution stage for motif updated dock score with RosettaDock 4.0 and ReplicaDock 2.0 for **medium docking targets.**
