# Supplementary material for: Induced fit with replica exchange improves protein complex structure prediction
Source: PLoS Comput Biol. 2022 Jun 3;18(6):e1010124. doi: 10.1371/journal.pcbi.1010124 (PMC9200320; doi:10.1371/journal.pcbi.1010124)
Supplement: S12 Fig — (PDF) [file pcbi.1010124.s015.pdf]

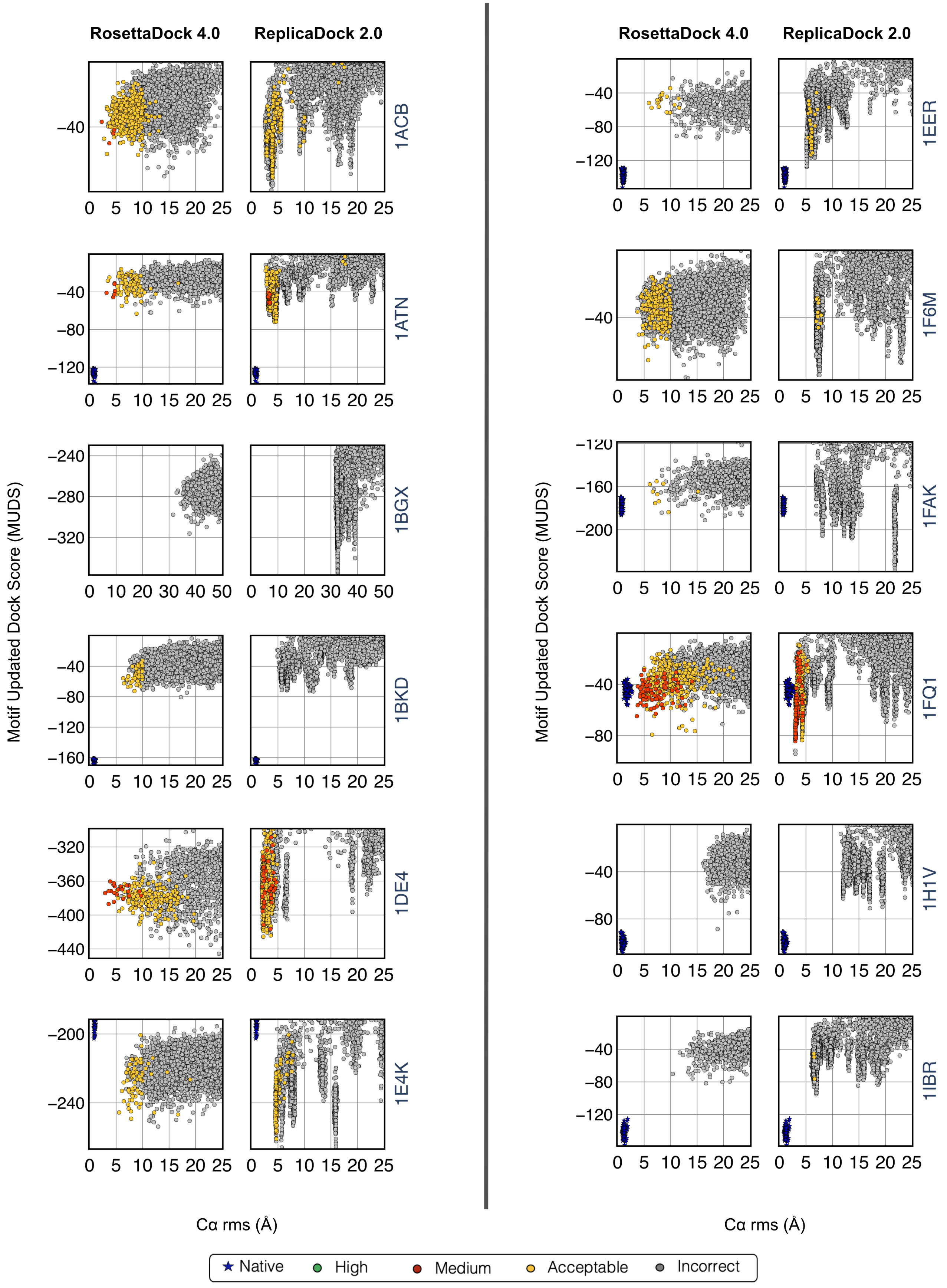

RosettaDock 4.0
ReplicaDock 2.0
1ACB
1ATN
1BGX
1BKD
1DE4
1E4K
1EER
1F6M
1FAK
1FQ1
1H1V
1IBR
Motif Updated Dock Score (MUDS)
Cα rms (Å)
Native
High
Medium
Acceptable
Incorrect

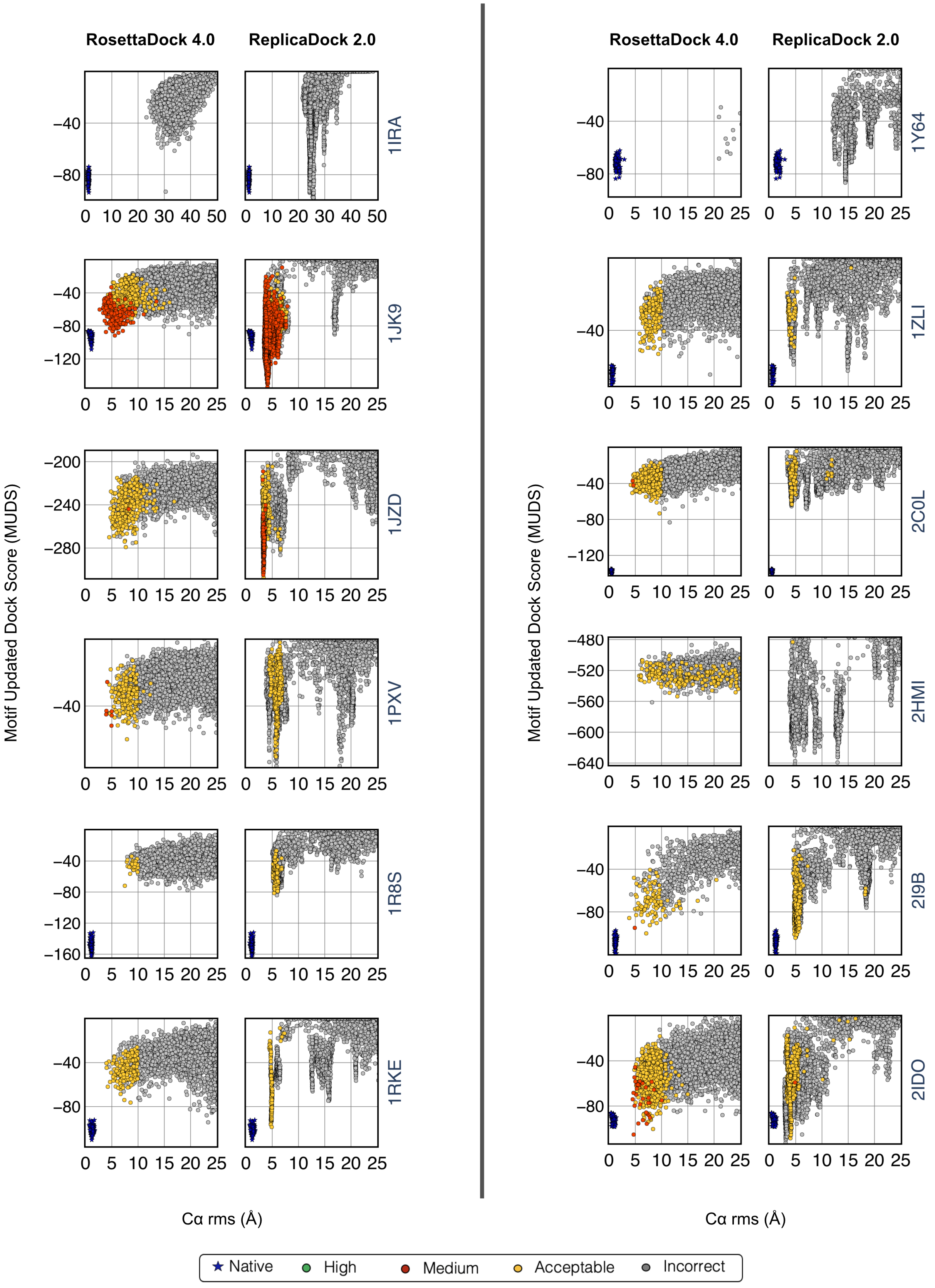

RosettaDock 4.0
ReplicaDock 2.0
1IRA
1JK9
1JZD
1PXV
1R8S
1RKE
1Y64
1ZLI
2C0L
2HMI
2I9B
2IDO
Motif Updated Dock Score (MUDS)
Cα rms (Å)
Native
High
Medium
Acceptable
Incorrect

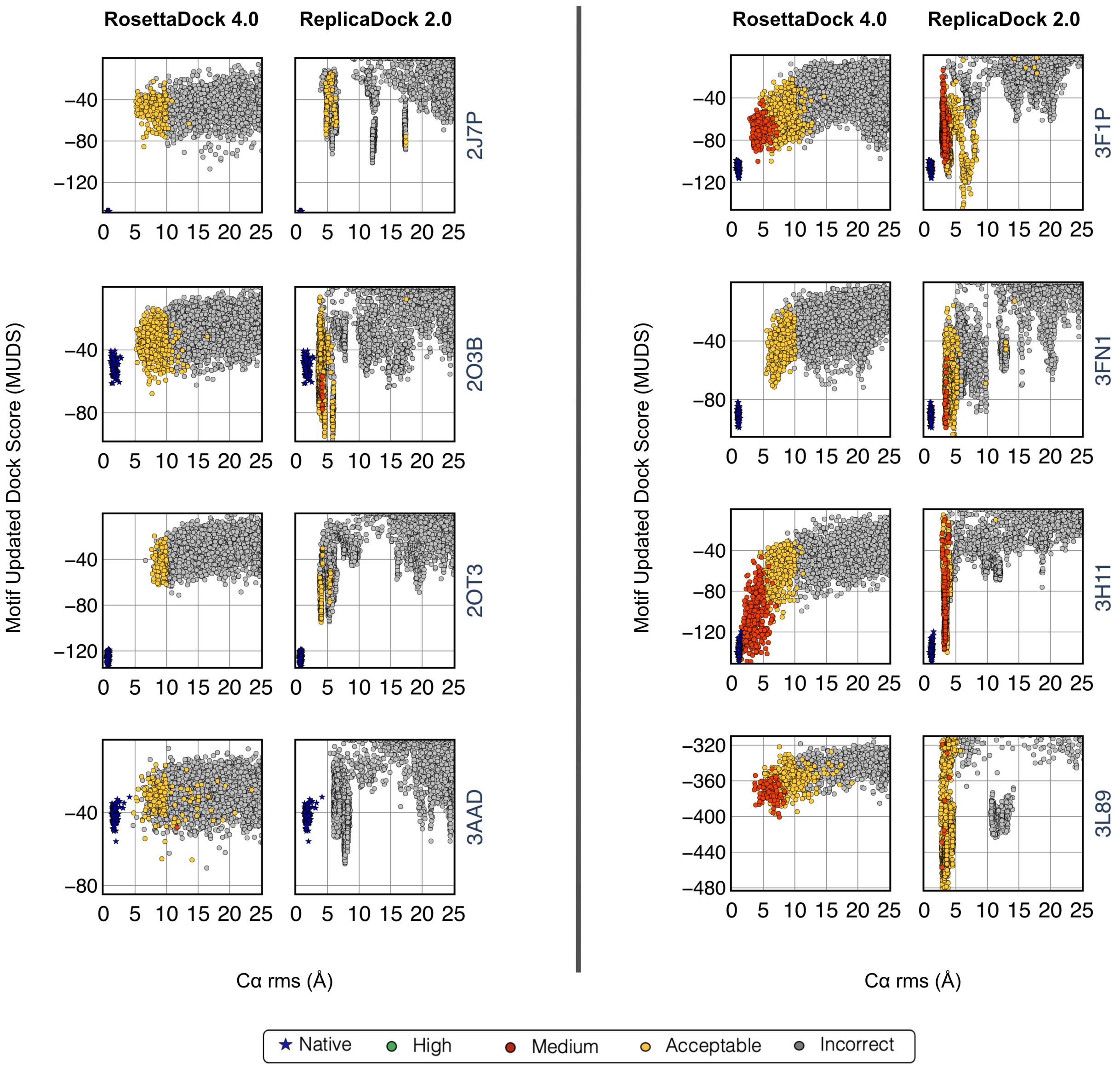

Fig. S12. Score versus Cα-RMSD(Å) plots in the low-resolution stage for motif updated dock score with RosettaDock 4.0 and ReplicaDock 2.0 for **difficult docking targets.**
