# Supplementary material for: Induced fit with replica exchange improves protein complex structure prediction
Source: PLoS Comput Biol. 2022 Jun 3;18(6):e1010124. doi: 10.1371/journal.pcbi.1010124 (PMC9200320; doi:10.1371/journal.pcbi.1010124)
Supplement: S13 Fig — (PDF) [file pcbi.1010124.s016.pdf]

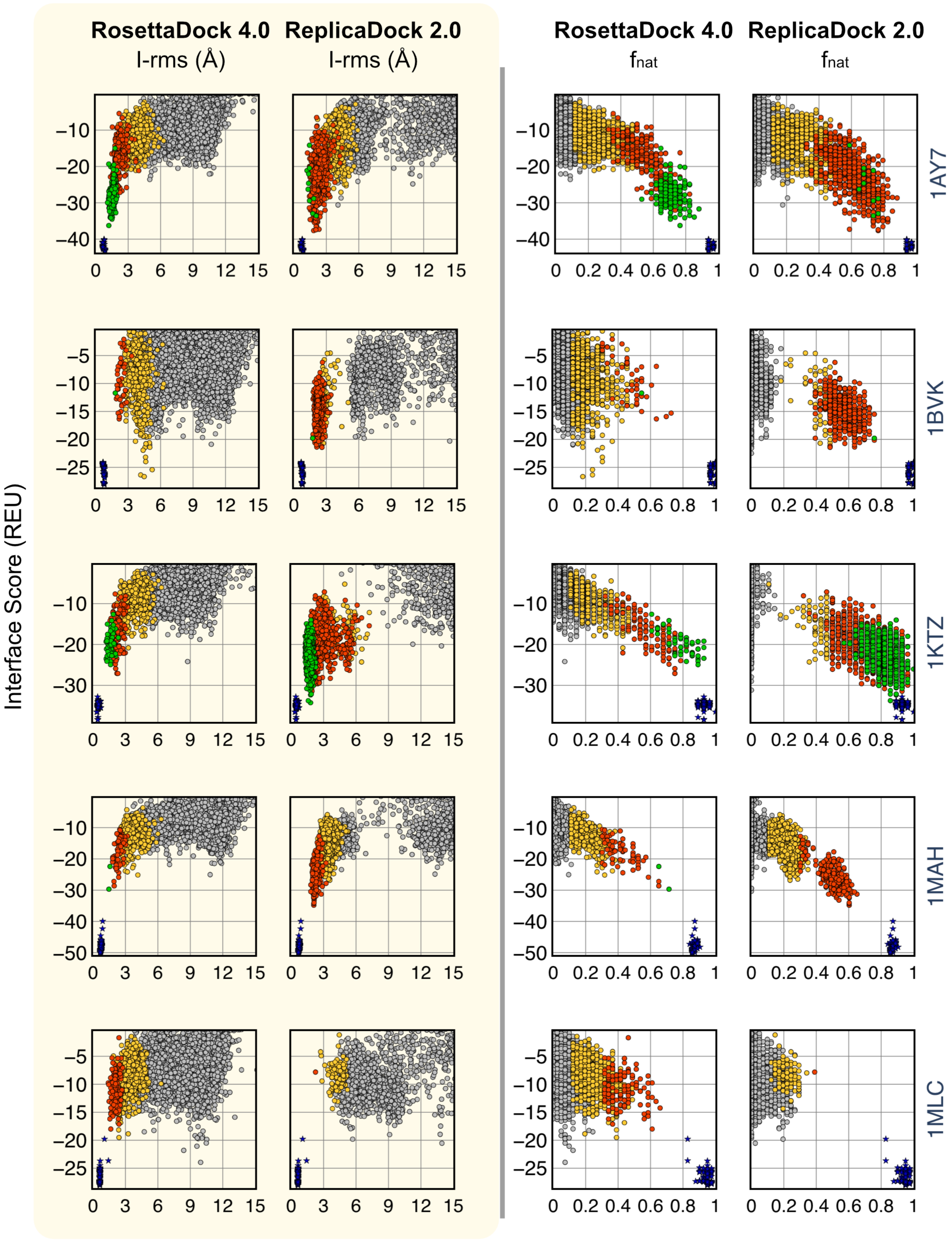

RosettaDock 4.0
ReplicaDock 2.0
I-rms (Å)
$f_{nat}$
Interface Score (REU)
1AY7
1BVK
1KTZ
1MAH
1MLC

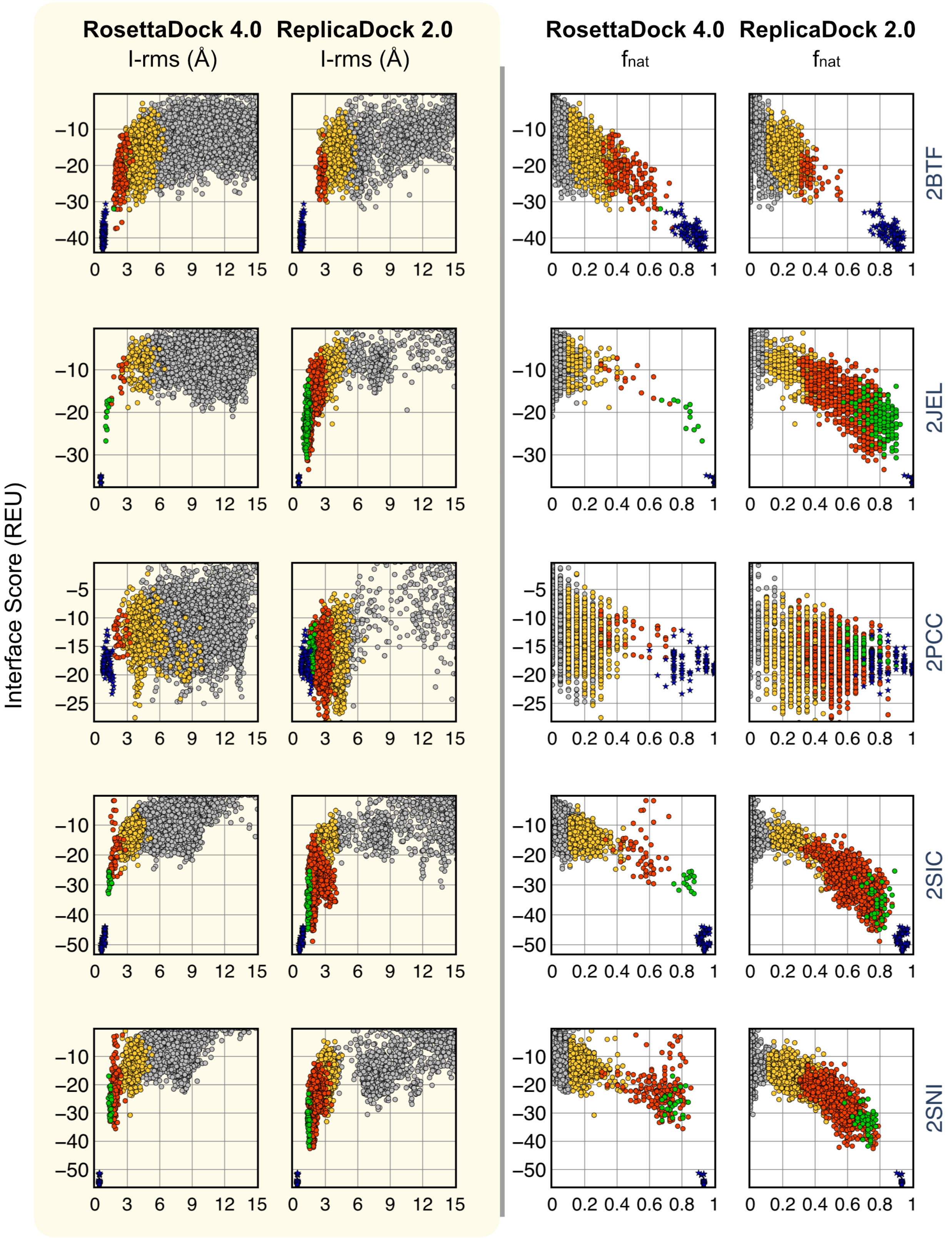


**Fig. S13.** Interface Score versus Interface-RMSD(Å) plots and Interface Score versus $f_{\mathrm{nat}}$ plots after the complete protocol for RosettaDock 4.0 and ReplicaDock 2.0 for rigid complexes.
