# Supplementary material for: Induced fit with replica exchange improves protein complex structure prediction
Source: PLoS Comput Biol. 2022 Jun 3;18(6):e1010124. doi: 10.1371/journal.pcbi.1010124 (PMC9200320; doi:10.1371/journal.pcbi.1010124)
Supplement: S14 Fig — (PDF) [file pcbi.1010124.s017.pdf]

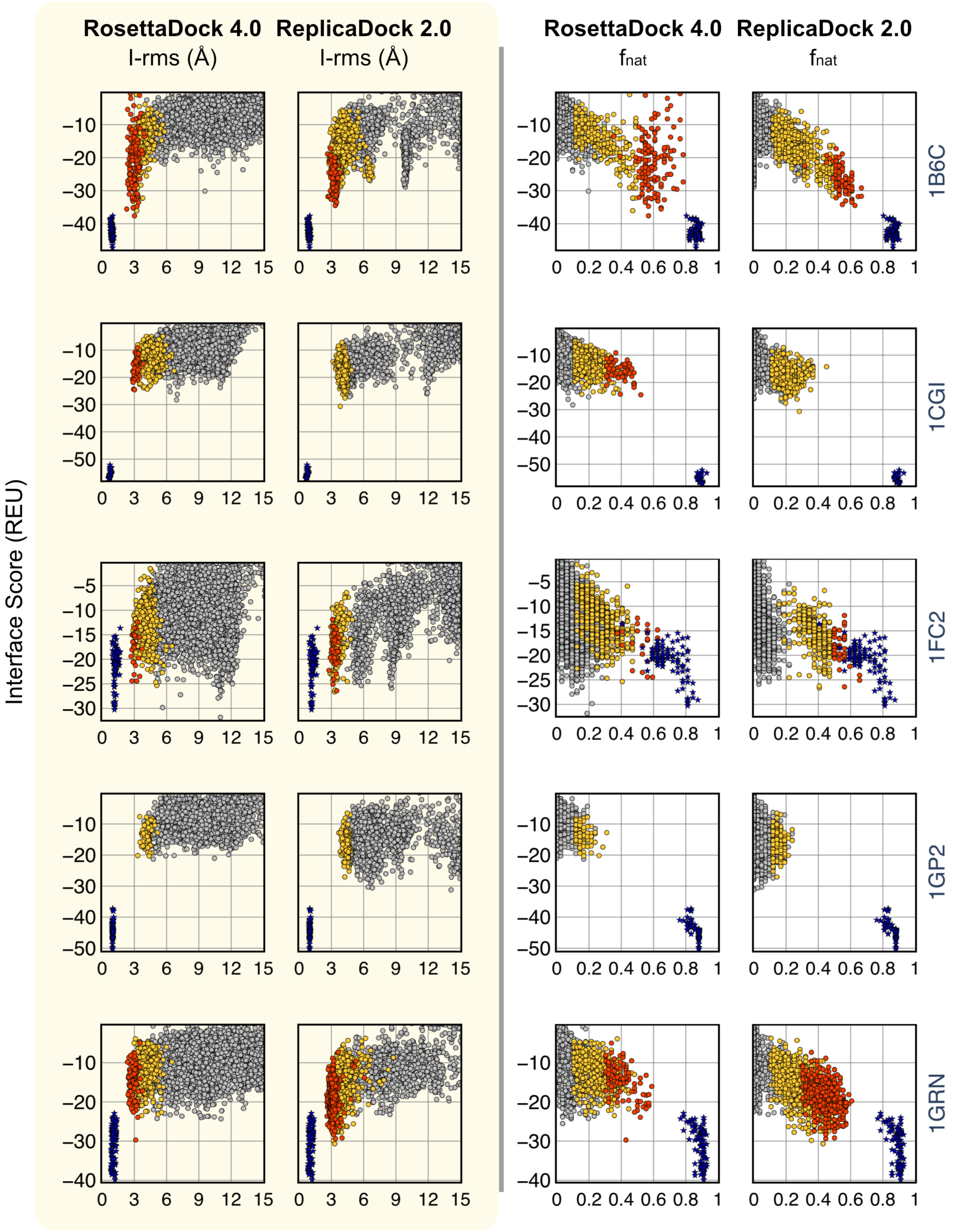
RosettaDock 4.0
ReplicaDock 2.0
I-rms (Å)
RosettaDock 4.0
ReplicaDock 2.0
f_{nat}
Interface Score (REU)
1B6C
1CGI
1FC2
1GP2
1GRN

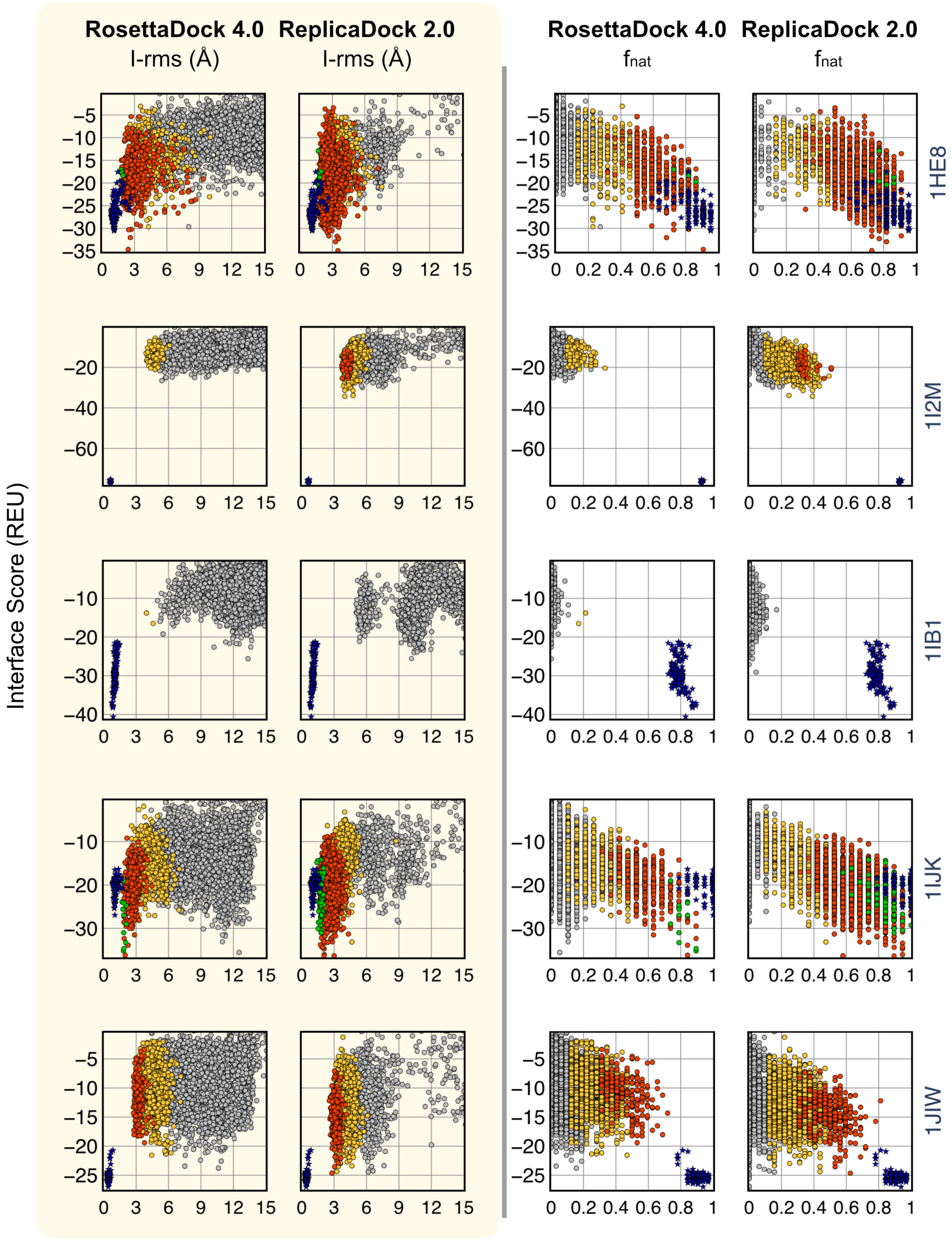

RosettaDock 4.0
I-rms (Å)
ReplicaDock 2.0
I-rms (Å)
RosettaDock 4.0
f_{nat}
ReplicaDock 2.0
f_{nat}
Interface Score (REU)
1HE8
1I2M
1IB1
1JJK
1JIW

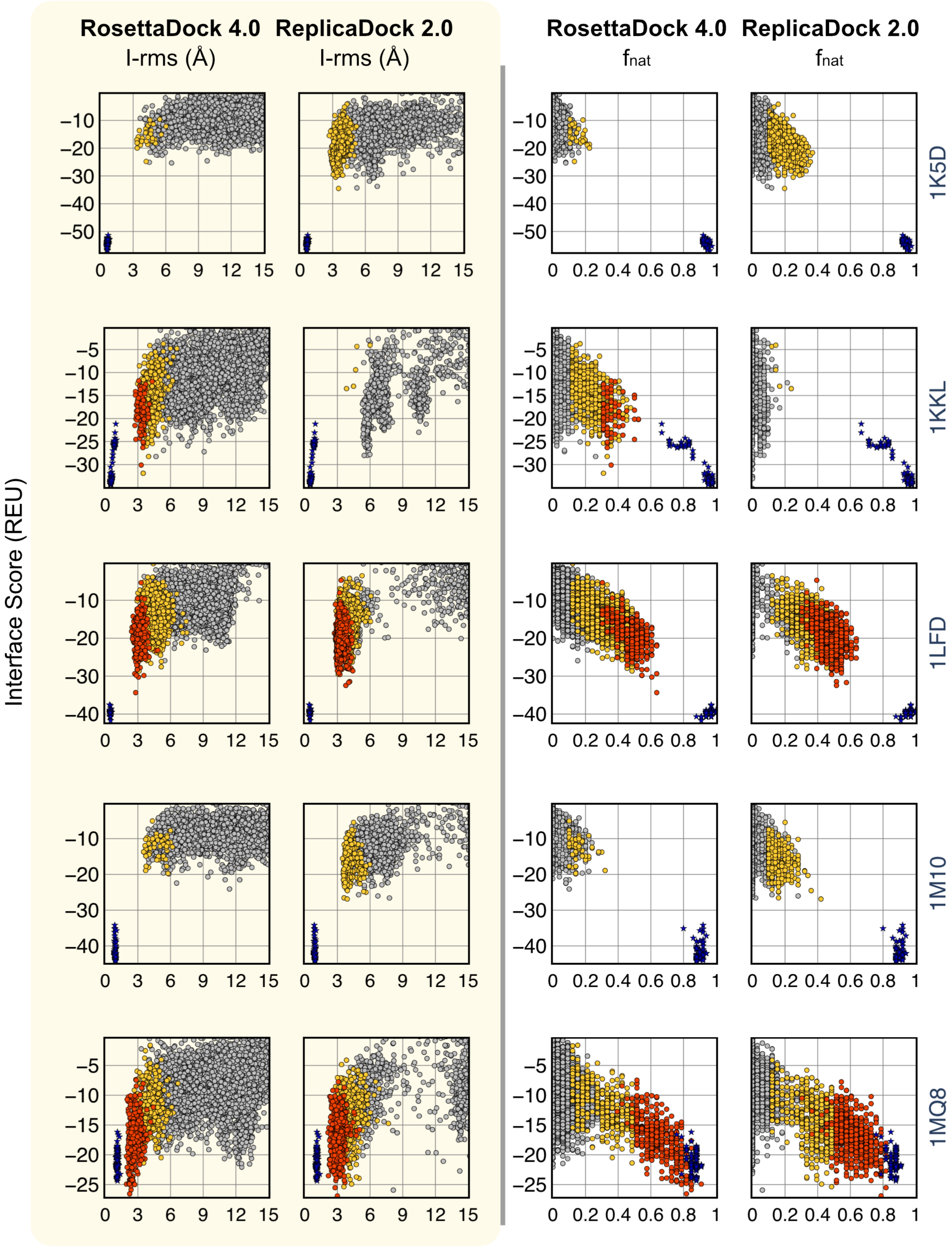

RosettaDock 4.0
ReplicaDock 2.0
I-rms (Å)
f_{nat}
Interface Score (REU)
1K5D
1KKL
1LFD
1M10
1MQ8

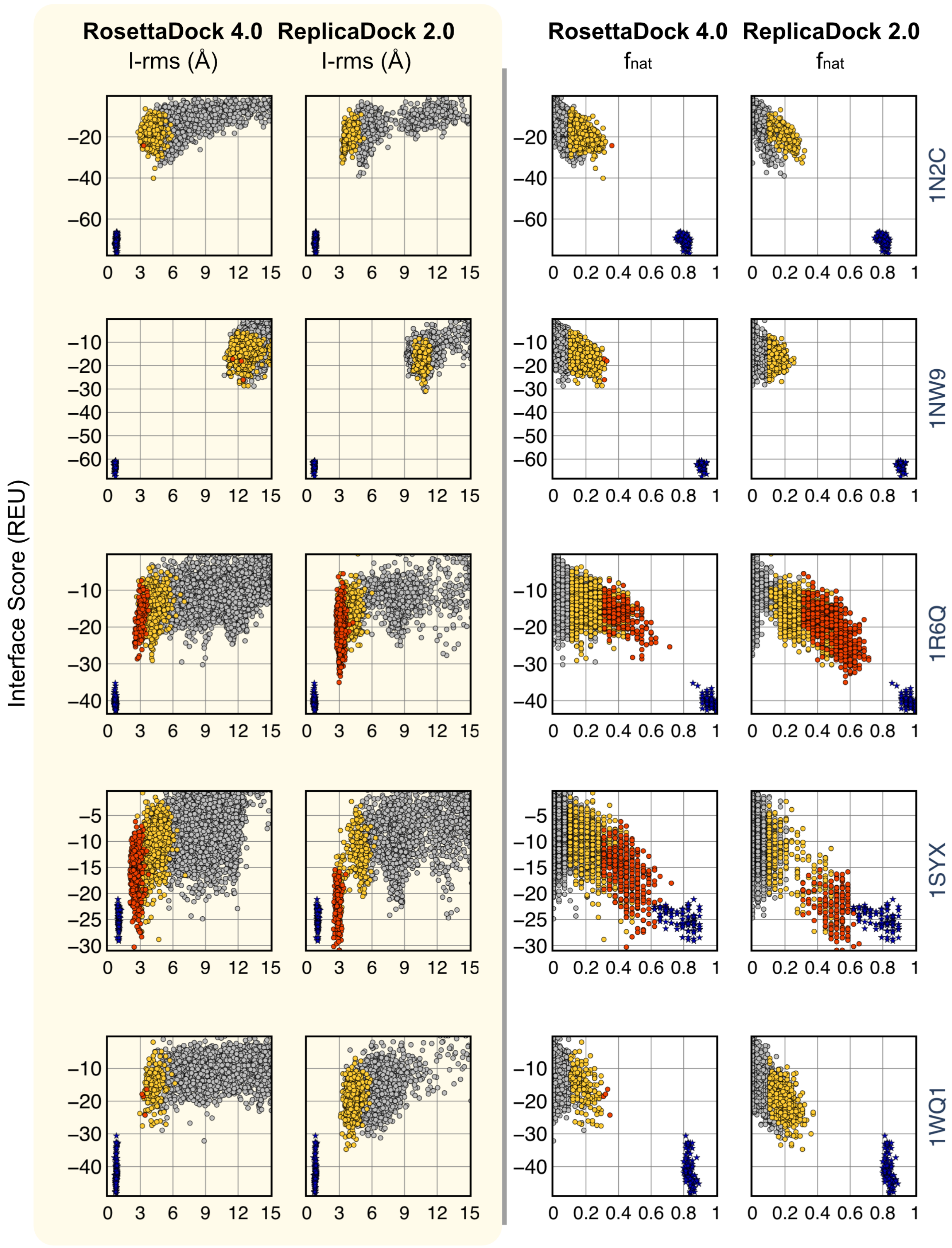
RosettaDock 4.0
ReplicaDock 2.0
I-rms (Å)
fnat
Interface Score (REU)
1N2C
1NW9
1R6Q
1SYX
1WQ1

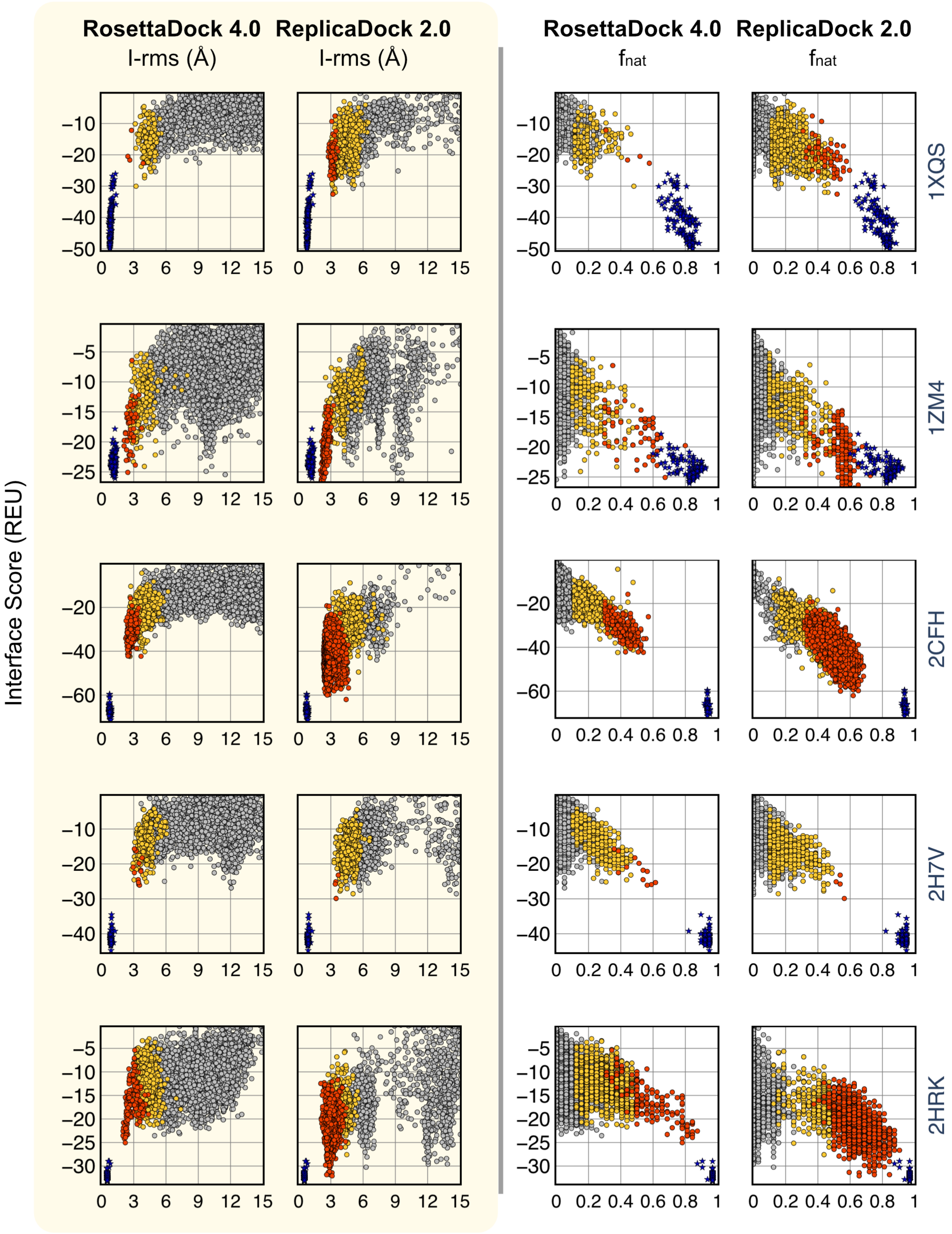

RosettaDock 4.0
ReplicaDock 2.0
I-rms (Å)
I-rms (Å)
RosettaDock 4.0
ReplicaDock 2.0
fnat
fnat
Interface Score (REU)
1XQS
1ZM4
2CFH
2H7V
2HRK

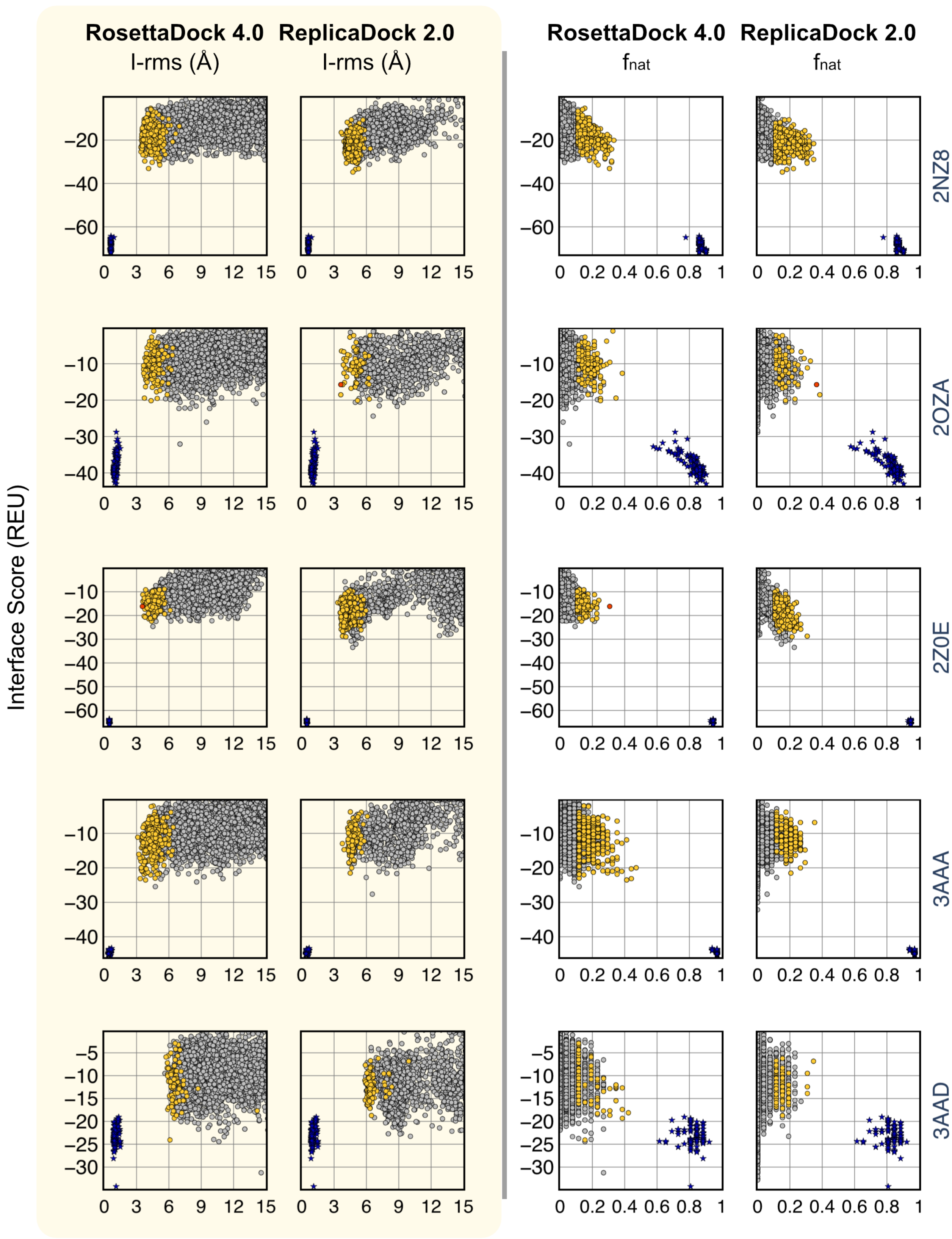

RosettaDock 4.0
ReplicaDock 2.0
I-rms (Å)
I-rms (Å)
RosettaDock 4.0
ReplicaDock 2.0
f_{nat}
f_{nat}
Interface Score (REU)
2NZ8
2OZA
2Z0E
3AAA
3AAD

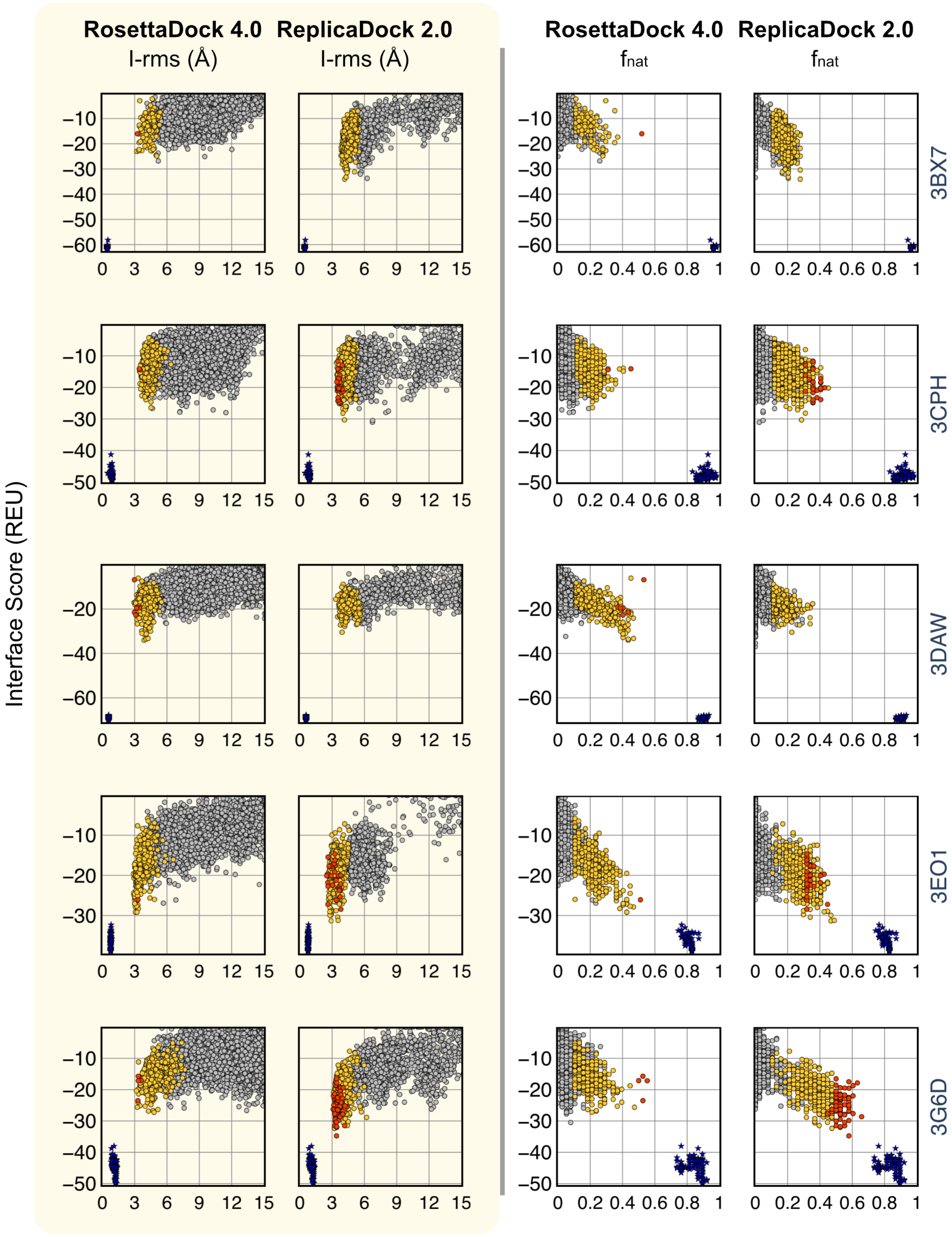
RosettaDock 4.0
I-rms (Å)
ReplicaDock 2.0
I-rms (Å)
RosettaDock 4.0
f_{nat}
ReplicaDock 2.0
f_{nat}
Interface Score (REU)
3BX7
3CPH
3DAW
3EO1
3G6D

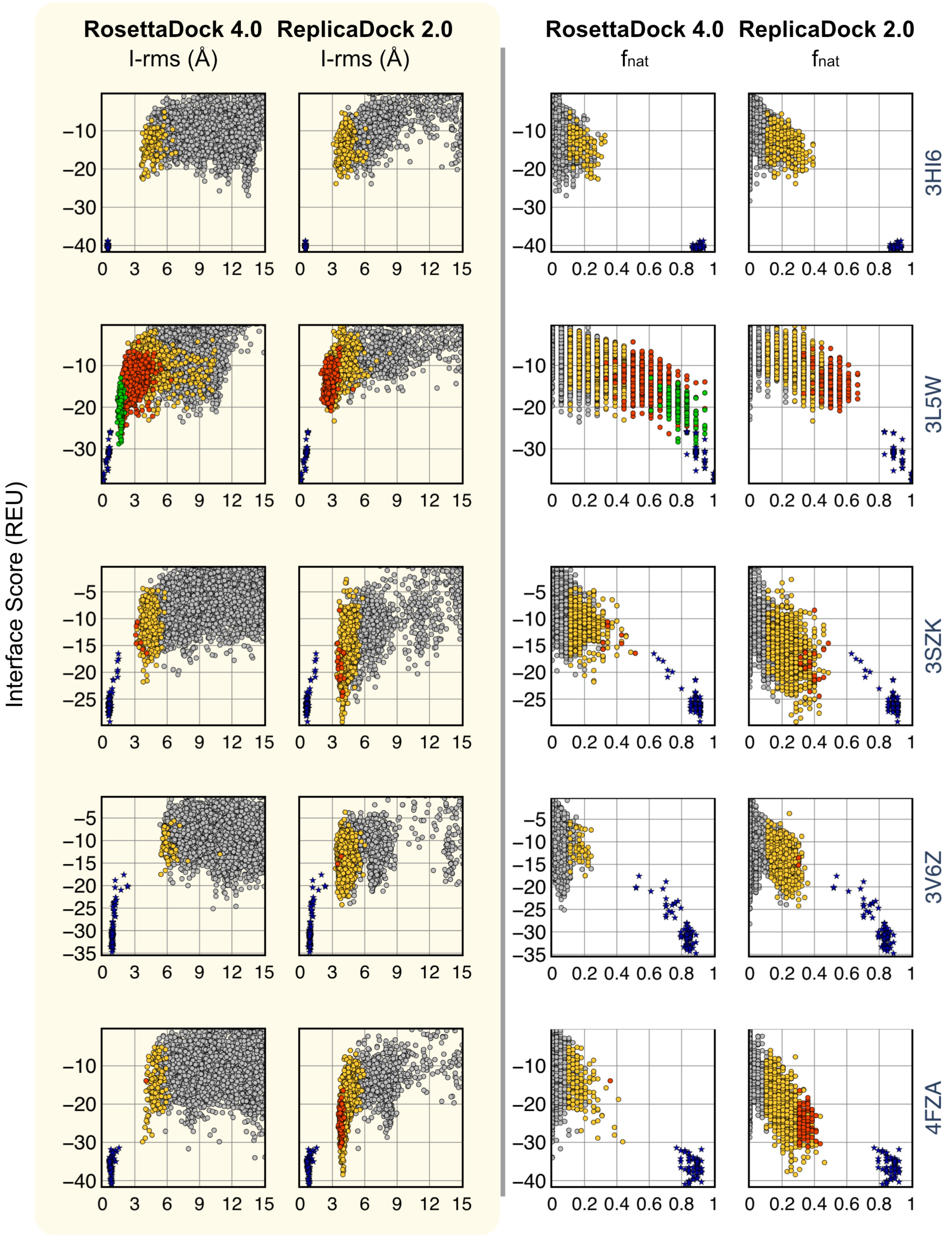

RosettaDock 4.0
ReplicaDock 2.0
I-rms (Å)
RosettaDock 4.0
ReplicaDock 2.0
f_{nat}
Interface Score (REU)
3HI6
3L5W
3SZK
3V6Z
4FZA

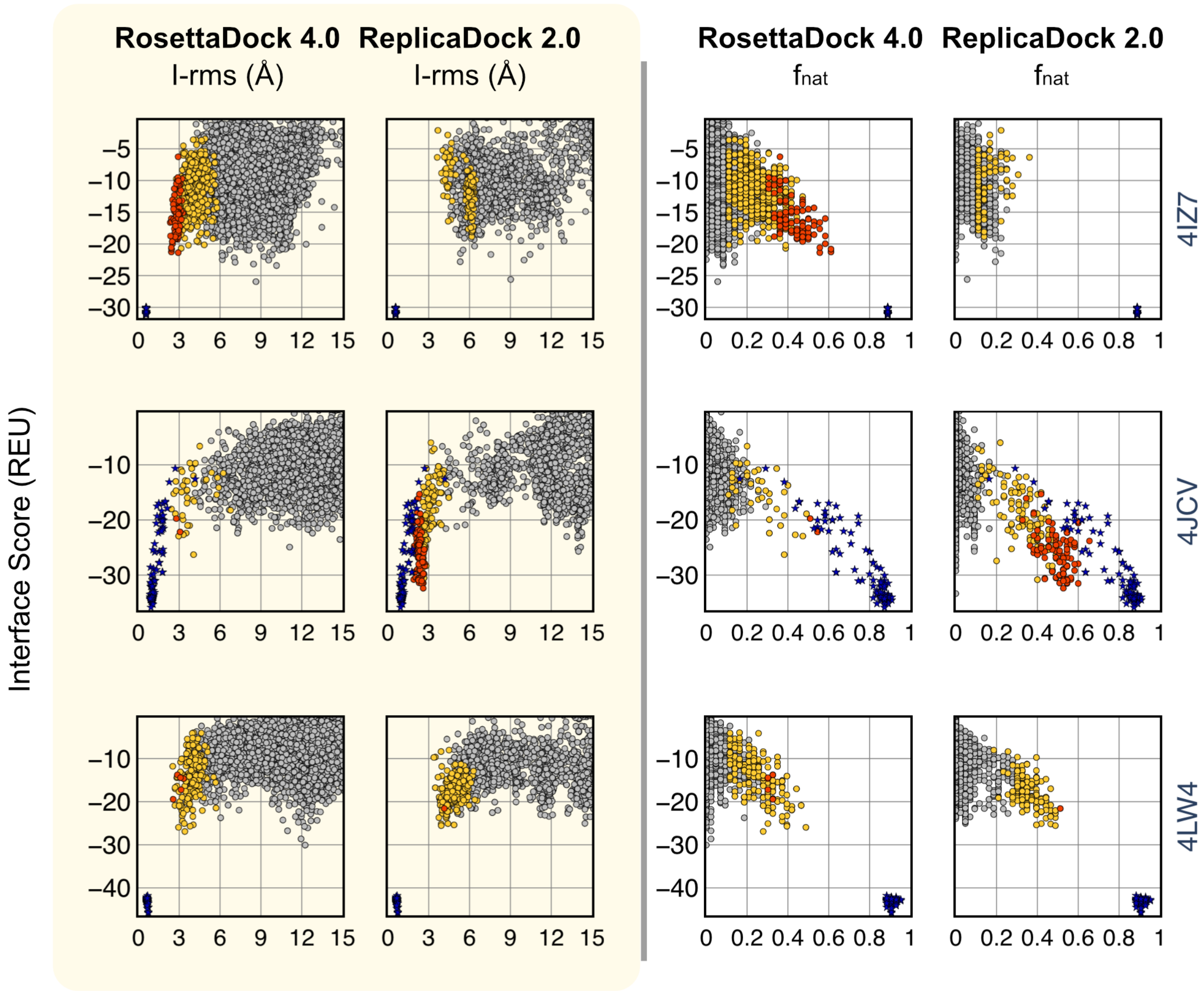

Fig. S14. Interface Score versus Interface-RMSD(Å) plots and Interface Score versus f_{nat} plots after the complete protocol for RosettaDock 4.0 and ReplicaDock 2.0 for **medium docking targets**.
