# Supplementary material for: Induced fit with replica exchange improves protein complex structure prediction
Source: PLoS Comput Biol. 2022 Jun 3;18(6):e1010124. doi: 10.1371/journal.pcbi.1010124 (PMC9200320; doi:10.1371/journal.pcbi.1010124)
Supplement: S15 Fig — (PDF) [file pcbi.1010124.s018.pdf]

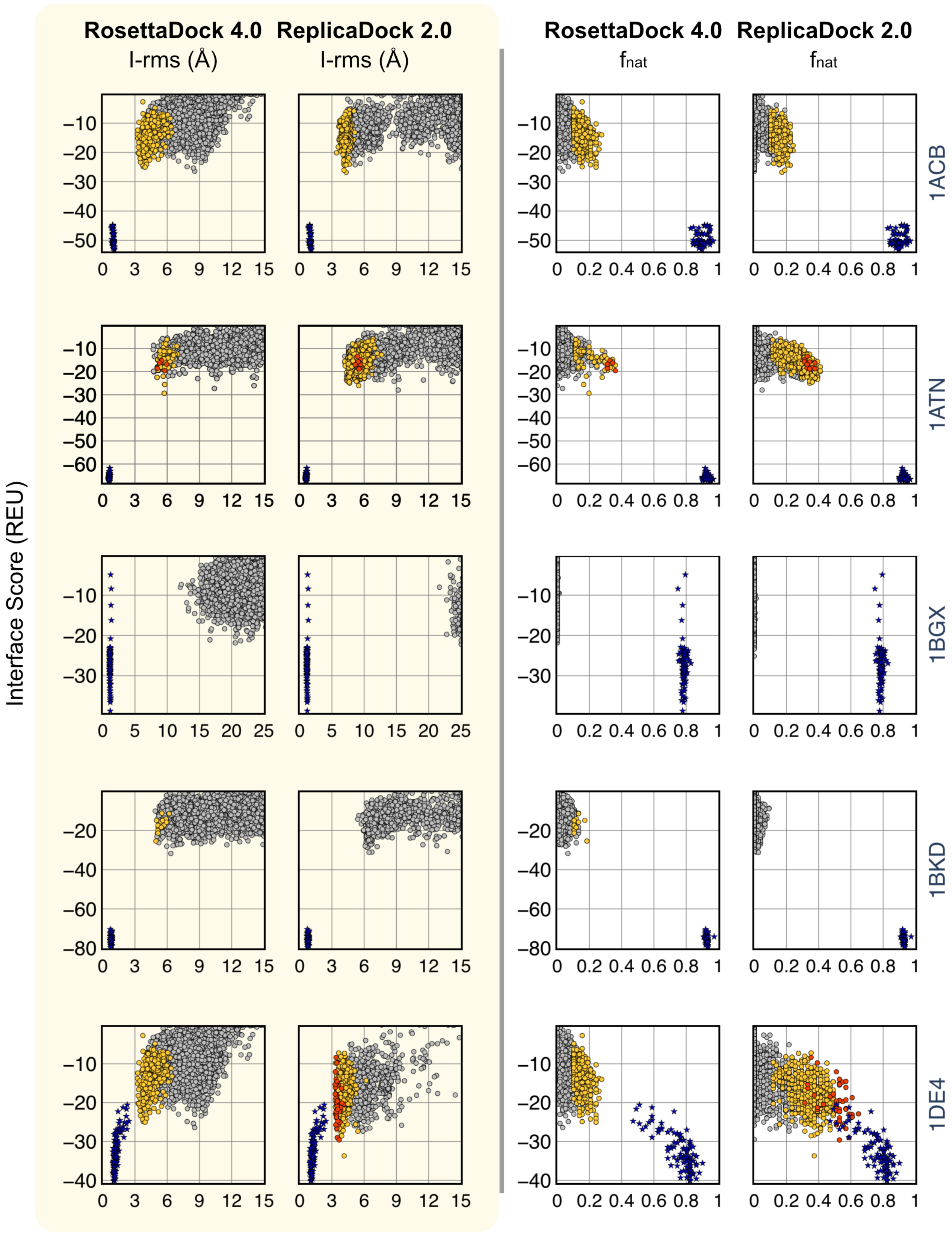

RosettaDock 4.0
ReplicaDock 2.0
I-rms (Å)
RosettaDock 4.0
ReplicaDock 2.0
f_{nat}
Interface Score (REU)
1ACB
1ATN
1BGX
1BKD
1DE4

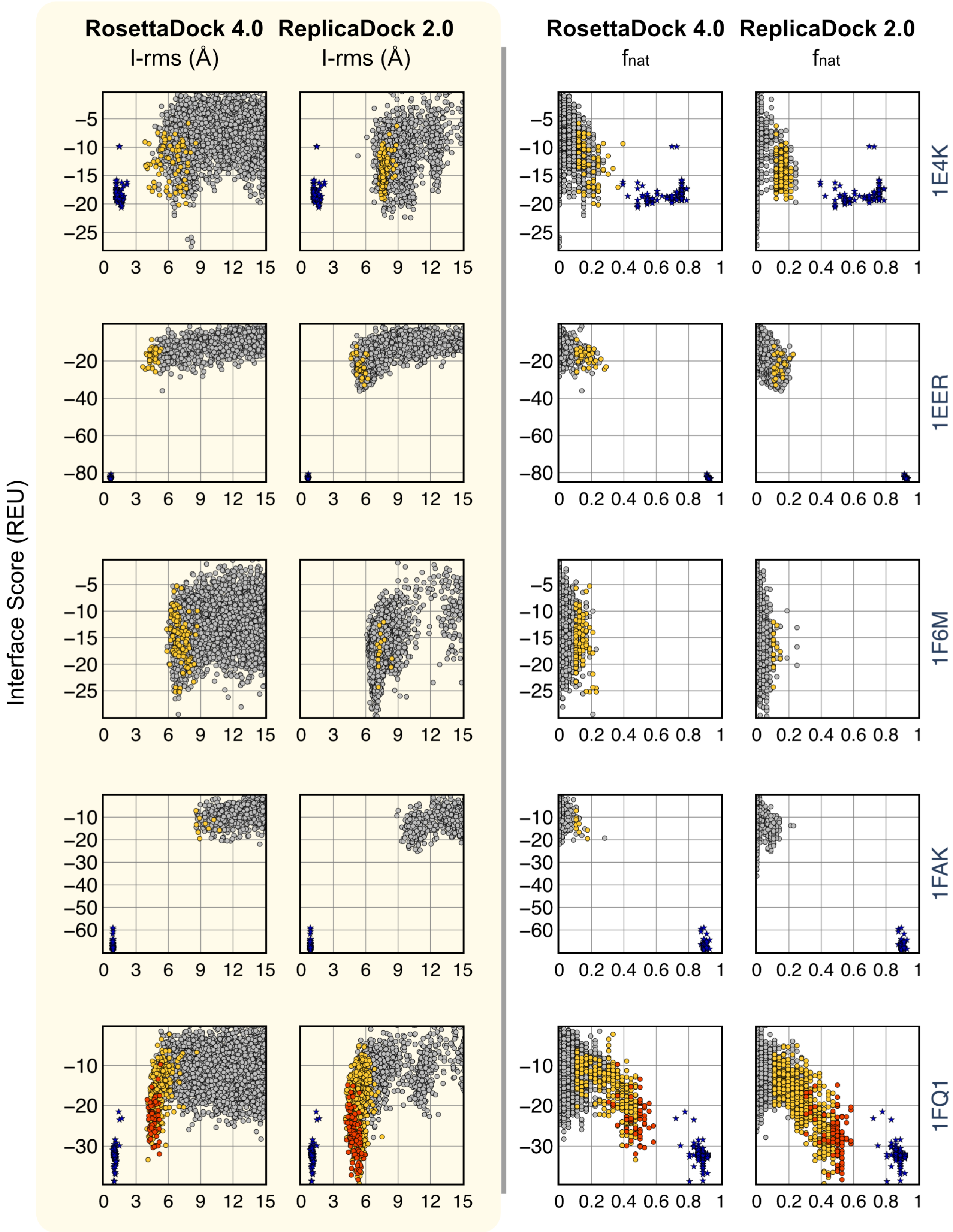

RosettaDock 4.0
I-rms (Å)
ReplicaDock 2.0
I-rms (Å)
RosettaDock 4.0
fnat
ReplicaDock 2.0
fnat
Interface Score (REU)
1E4K
1EER
1F6M
1FAK
1FQ1

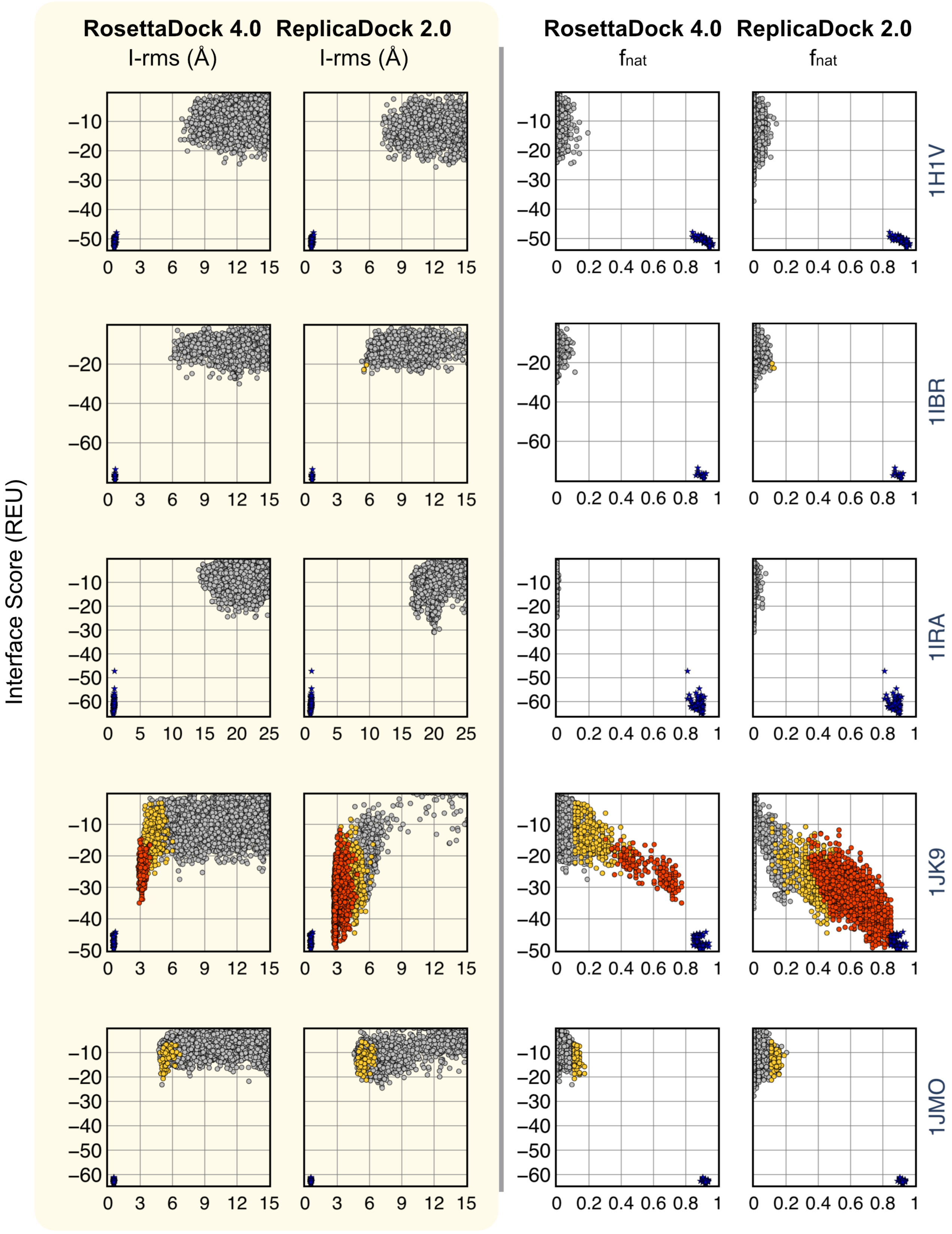
RosettaDock 4.0
ReplicaDock 2.0
I-rms (Å)
RosettaDock 4.0
ReplicaDock 2.0
f_{nat}
Interface Score (REU)
1H1V
1IBR
1IRA
1JK9
1JMO

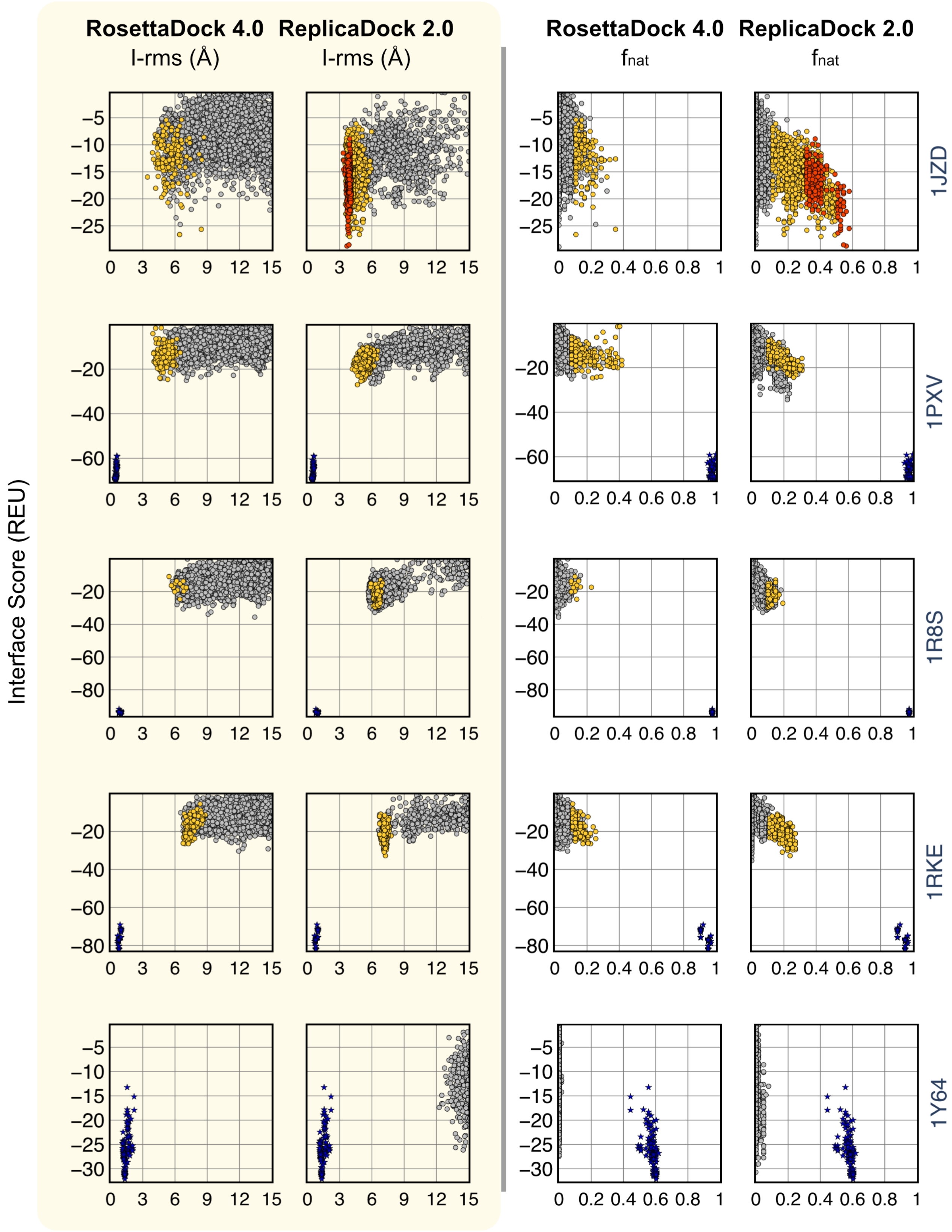
RosettaDock 4.0
ReplicaDock 2.0
I-rms (Å)
RosettaDock 4.0
ReplicaDock 2.0
f_{nat}
Interface Score (REU)
1JZD
1PXV
1R8S
1RKE
1Y64

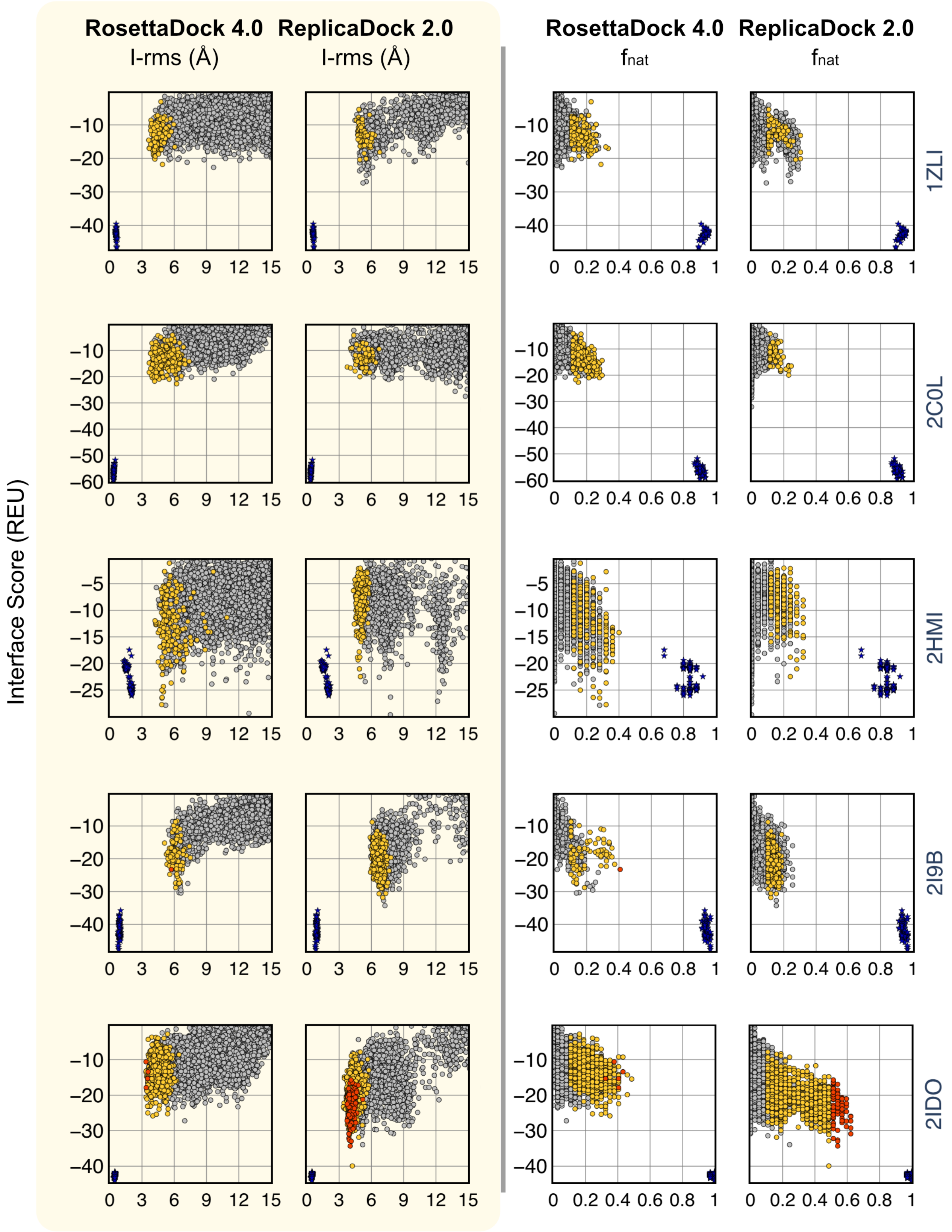
RosettaDock 4.0
ReplicaDock 2.0
I-rms (Å)
RosettaDock 4.0
ReplicaDock 2.0
fnat
Interface Score (REU)
1ZLI
2C0L
2HMI
2I9B
2IDO

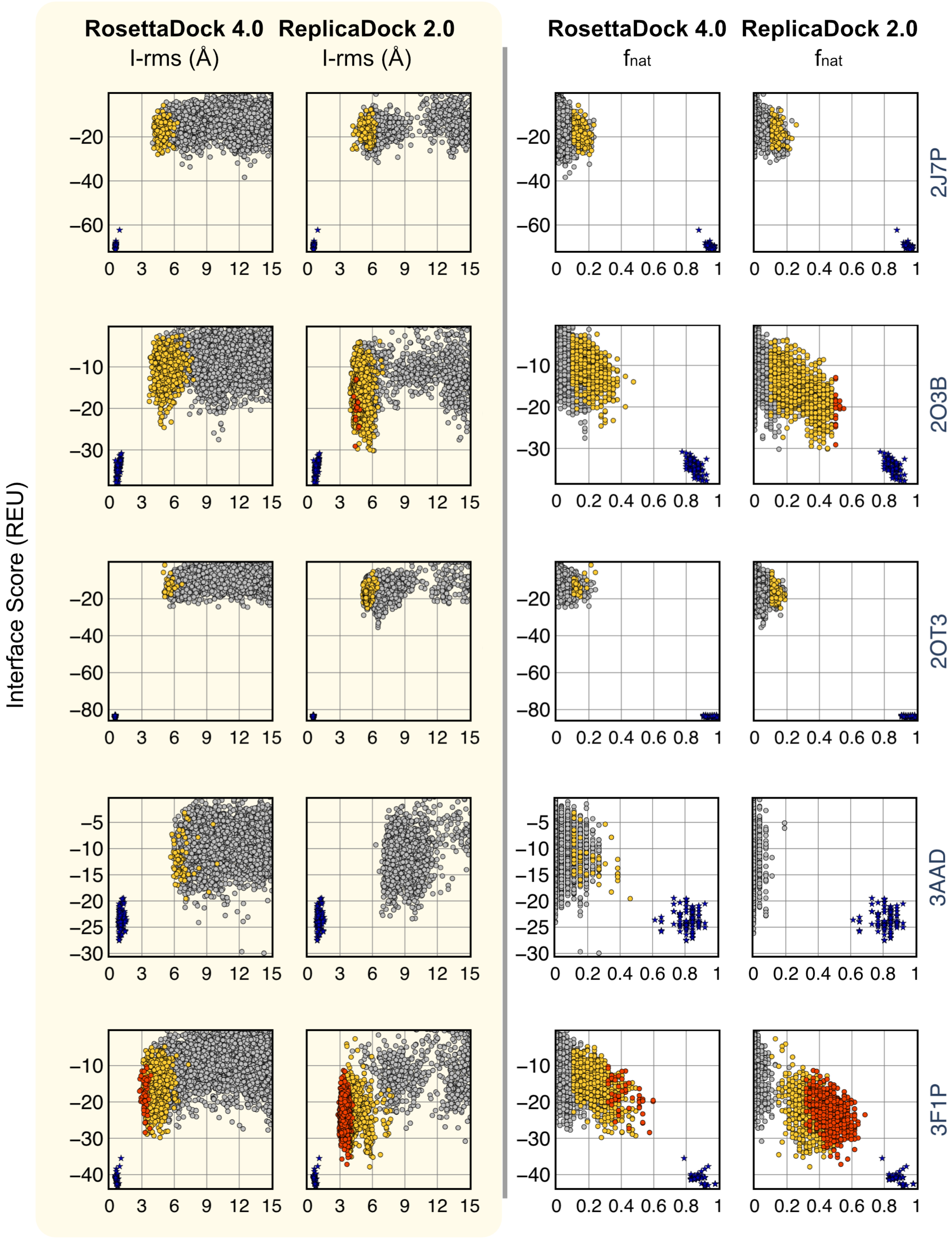
RosettaDock 4.0
ReplicaDock 2.0
I-rms (Å)
fnat
Interface Score (REU)
2J7P
2O3B
2OT3
3AAD
3F1P

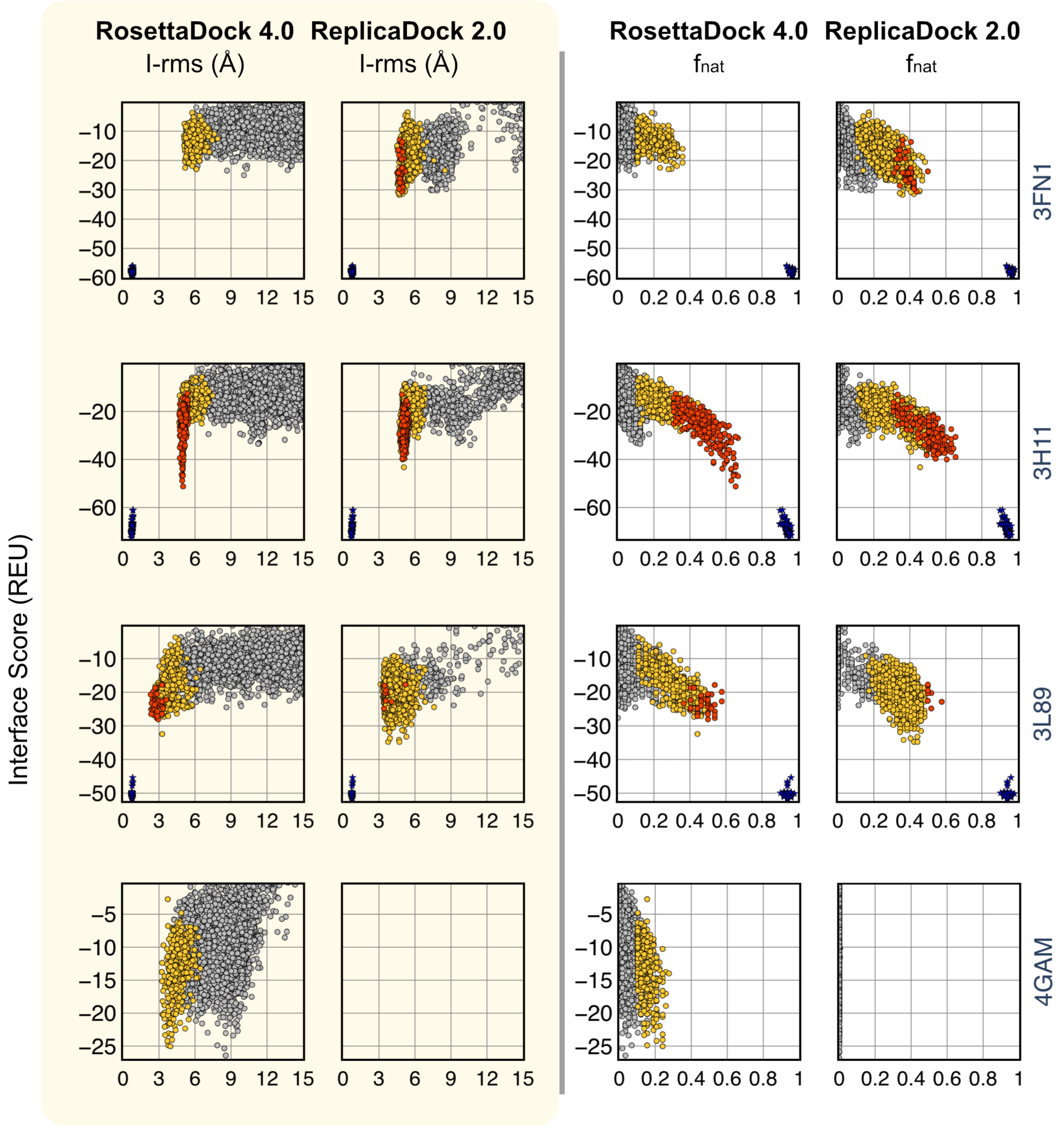

Fig. S15. Interface Score versus Interface-RMSD(Å) plots and Interface Score versus f_{nat} plots after the complete protocol for RosettaDock 4.0 and ReplicaDock 2.0 for **difficult docking targets**.
