# Supplementary material for: Induced fit with replica exchange improves protein complex structure prediction
Source: PLoS Comput Biol. 2022 Jun 3;18(6):e1010124. doi: 10.1371/journal.pcbi.1010124 (PMC9200320; doi:10.1371/journal.pcbi.1010124)
Supplement: S16 Fig — (PDF) [file pcbi.1010124.s019.pdf]

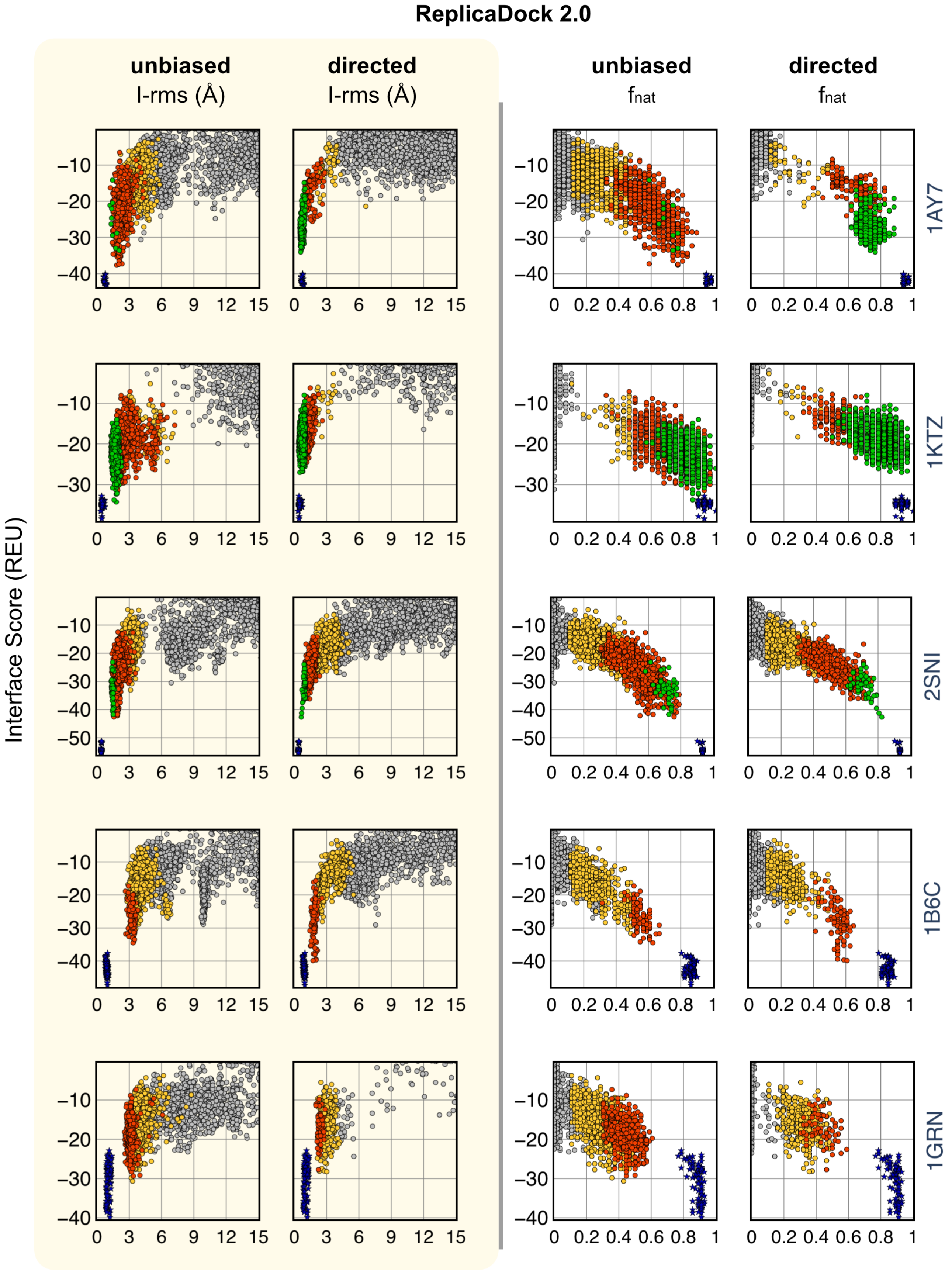
ReplicaDock 2.0
unbiased
I-rms (Å)
directed
I-rms (Å)
unbiased
fnat
directed
fnat
Interface Score (REU)
1AY7
1KTZ
2SNI
1B6C
1GRN

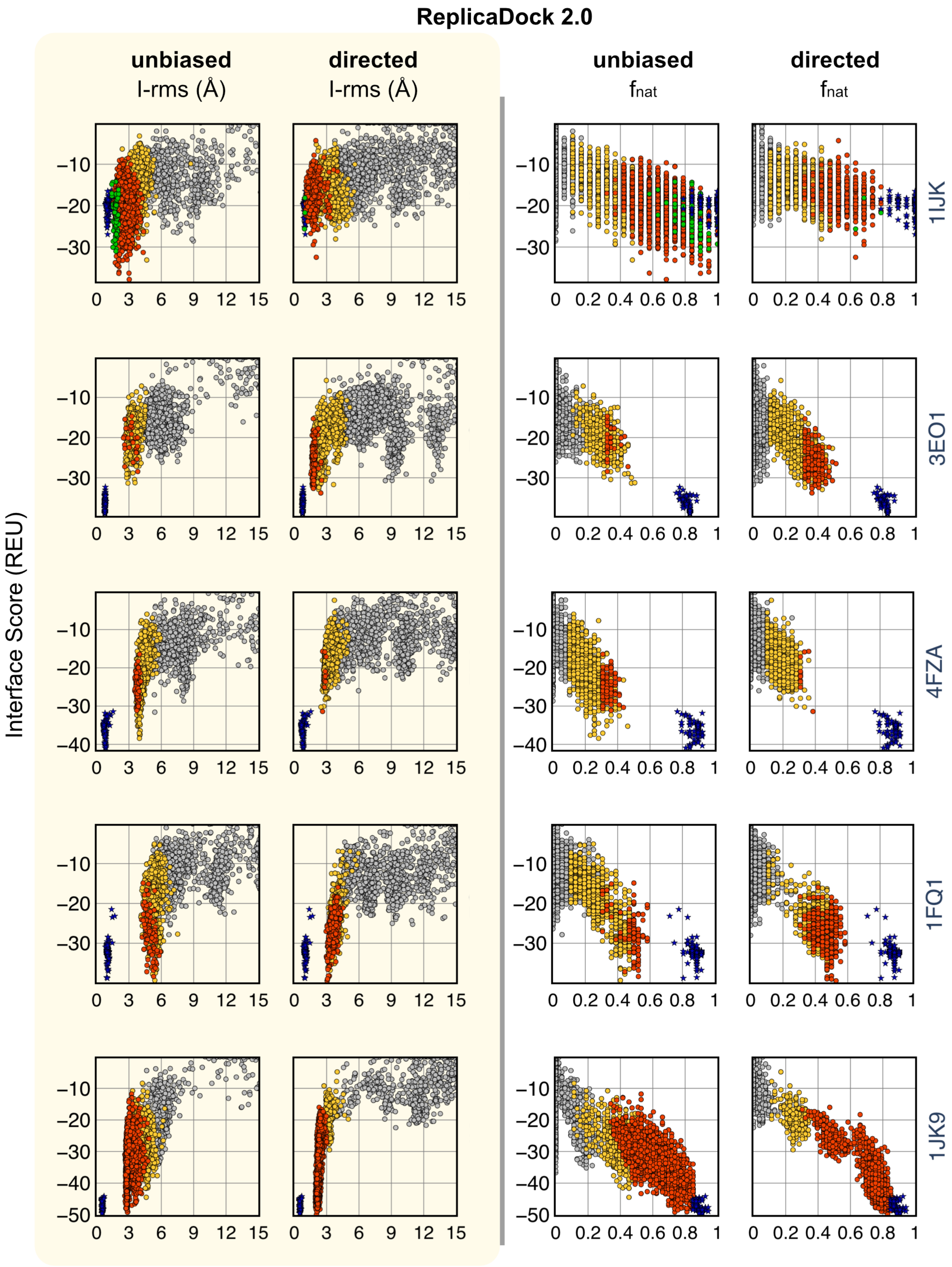

ReplicaDock 2.0
unbiased
I-rms (Å)
directed
I-rms (Å)
unbiased
f_{nat}
directed
f_{nat}
Interface Score (REU)
1IJK
3EO1
4FZA
1FQ1
1JK9

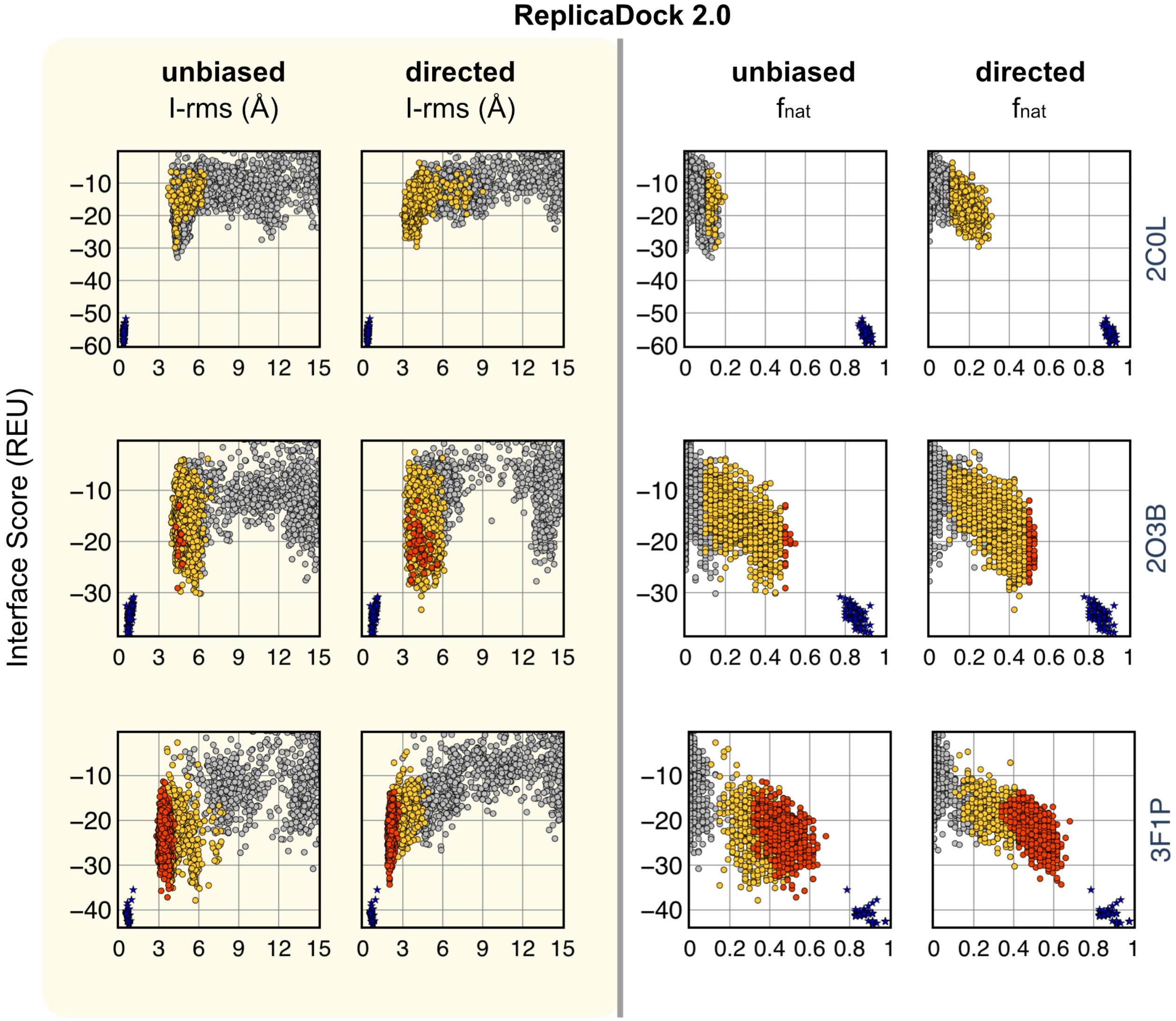

Fig. S16. Interface Score versus Interface-RMSD(Å) plots and Interface Score versus f_{nat} plots after the complete protocol with RosettaDock 4.0 and with directed induced-fit sampling for ReplicaDock 2.0 for benchmark 5.0 targets.
