# Supplementary material for: Induced fit with replica exchange improves protein complex structure prediction
Source: PLoS Comput Biol. 2022 Jun 3;18(6):e1010124. doi: 10.1371/journal.pcbi.1010124 (PMC9200320; doi:10.1371/journal.pcbi.1010124)
Supplement: S17 Fig — Scaling of docking simulations on XSEDE’s Rockfish Cluster for protein docking targets from the DB5.5 with respect to the number of residues. (PDF) [file pcbi.1010124.s020.pdf]

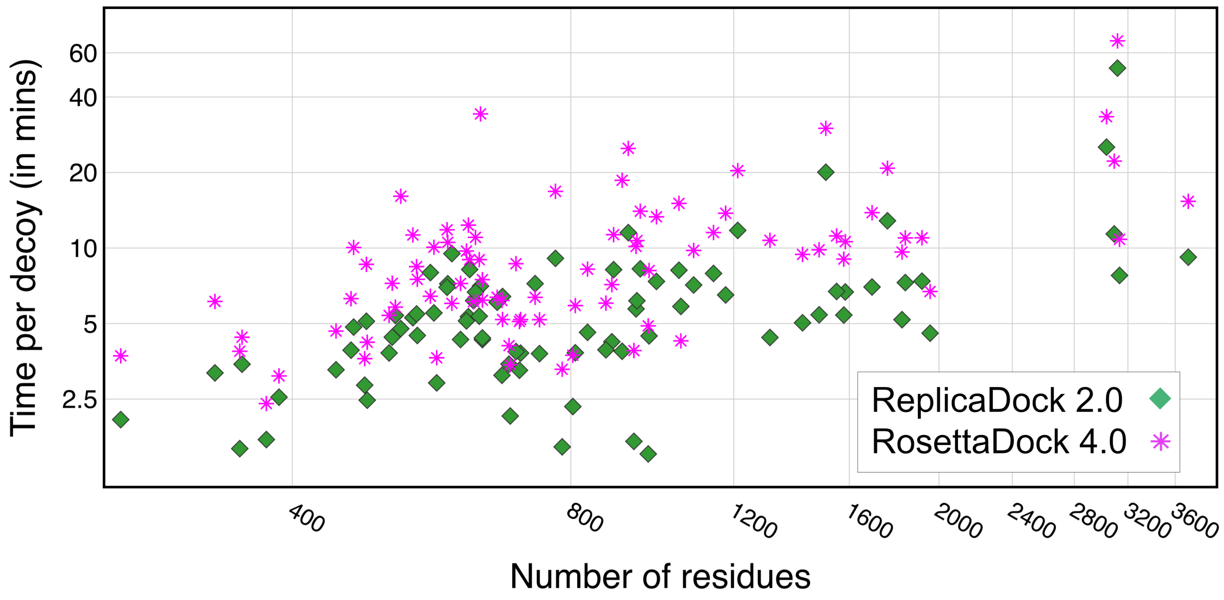

Fig. S17. Compute time comparison between ReplicaDock2.0 and RosettaDock4.0. Scaling of docking simulations on XSEDE's Rockfish Cluster for protein docking targets from the DB5.5 with respect to the number of residues.
