# Supplementary material for: Induced fit with replica exchange improves protein complex structure prediction
Source: PLoS Comput Biol. 2022 Jun 3;18(6):e1010124. doi: 10.1371/journal.pcbi.1010124 (PMC9200320; doi:10.1371/journal.pcbi.1010124)
Supplement: Attachment — Submitted filename: review_OSF_NA.pdf [file pcbi.1010124.s021.pdf]

Review

Summary:

The ReplicaDock 2.0 protein-protein docking method introduced in this paper is aimed at the modeling of conformational changes induced by the binding partner (induced fit). In a previous effort, the Gray lab has introduced RosettaDock4.0 that addressed conformational flexibility as conformer ensembles. Here, the focus is put on binding-induced conformational changes. ReplicaDock 2.0 builds on replica-exchange MC-based (REMC) rigid-docking protocol (ReplicaDock, developed by Zhang et al.). The authors used an improved low-resolution scoring function (MUDS: Motif Dock Score adapted to include backbone movements) and introduced backbone sampling into REMC (using backrub moves).

The protocol is assessed in the following way: first, rigid global docking was performed on a set of 10 protein targets starting from random orientations of the protein partners. The new low-resolution scoring function was shown to improve the sampling to identify local binding patches. Overall however, the improvements compared to RosettaDock4.0 in global docking are not very dramatic. It seems to improve fnat in global docking in a few challenging cases with medium-to-highly flexible backbones. It is computation intensive (e.g., 20x cluspro). Importantly, however, comparison of RosettaDock4.0 to ReplicaDock2.0 allows us to assess the contribution of conformer selection vs induced fit.

Second, flexible local docking on a set of 12 complexes showed deeper sampling of energy funnels. The flexibility of the receptor backbone was optimized (use of an 8 Å interface patch was found to be optimal). However, despite generating lower energy structures, the improvement towards the native structure seems marginal.

Lastly, for a large benchmark set of 88 complexes, the protocol improved predictions compared to RosettaDock4.0, for rigid, medium as well as flexible cases. However, improvement is still marginal, and for highly flexible complexes, sampling is not sufficient. This was improved with targeted sampling, when the flexible region (e.g. loop) is known.

Given the marginal improvement of the blind protocol, contrasted to the significant improvement once the flexible regions are known, it is clear that one needs to investigate methods to identify these regions. Such possible directions are mentioned in the discussion section.

Overall, this study presents an important step forward in the challenging path of flexible docking. Methods and results are presented clearly and in detail. I have only a few questions, and comments that could be addressed to improve the manuscript.

Major suggestion:

1. One topic that could be discussed more is the marginal improvement of high-resolution refinement. Is it indeed necessary, after introducing MDS (and MUDS)?

Other suggestions:

2. The protocol does not include any “prepacking” step, in which internal clashes of the receptor and ligand are removed before the start of the docking procedure. If backbone changes lead to changes in internal energy, then a docking trajectory could in principle result in different local relaxations that affect the evaluation score. Could the authors comment on this?
3. For global docking, a constant number of decoy trajectories are sampled (n=24k). Does this affect performance for larger complexes? Could scaling this based on the receptor surface improve efficiency and performance?

4. Also related to sampling (Page 14): for generating starting conformations for local refinement, it seems that the perturbation involves only translation on the axis that connects the two centers of mass (moving 15A away), but no translation in the other two dimensions. Please clarify that this is the case, and discuss whether this indeeds warrants random local sampling.

5. Clustering: the results are presented without clustering, leading to cases where we have either N5=5 or N5=0 (at least in the low resolution step). In cases of N5=0, would clustering help?

6. From the figure, it is not clear how much the high resolution step improves the results. Can the authors discuss this more? In addition, could maybe bb minimization at the high res step help? Or would it just increase runtime?

Technical details:

7. References: Move reference #2 from line 9 to line 13 (is about flexible docking, less about PPI networks). The supplementary material is missing the bibliography.

8. FIgure 3B,E: include as “x” the starting RMSD of the receptor and ligand - will allow to assess whether we move into the right direction

9. FIgure 4A: define the three numbers included at the top of the figure explicitly (better, equally and worse-modeled complexes).

10. Figure 4B: the density plots of the dark green and light green lines (ReplicaDock2 high res and low res) don’t cover the same area: how come?

11. In Table S2, highlight the pdbs that are included in the n=12 dataset in Fig 2B and n=11 in Figure S6.

12. Figure S3: Please define the label “Gray”. I assume it refers to ReplicaDock2 reported here.

13. Figure S6: label (a) and (b), or indicate “left” and “right”
