# Supplementary material for: Induced fit with replica exchange improves protein complex structure prediction
Source: PLoS Comput Biol. 2022 Jun 3;18(6):e1010124. doi: 10.1371/journal.pcbi.1010124 (PMC9200320; doi:10.1371/journal.pcbi.1010124)
Supplement: Attachment — Submitted filename: Responses_Harmalkar2021.pdf [file pcbi.1010124.s022.pdf]

To the editors:

I am pleased to submit a revision of our manuscript entitled “Induced fit with replica exchange improves protein complex structure prediction” by Ameya Harmalkar, Sai Pooja Mahajan and myself, in *PLOS Computational Biology*. We want to thank the reviewers and editors for their valuable feedback and comments. We have addressed all the comments from the reviewers (see following pages) and have made appropriate changes in our manuscript. Our responses to the reviewer comments (*highlighted in blue*) with the description of changes in the manuscript (*highlighted in green*) are presented below. The changes in the manuscript (PDF version) are highlighted in red.

Thank you for your consideration of this manuscript. We look forward to your reply.

Reviewer #1:

In this manuscript the authors introduce ReplicaDock 2.0, that continues the previous work on replica-exchange MC-based rigid body docking, implementing backbone flexibility and improving the scoring function. This approach aims to tackle the conformational changes via induced fit.

1. For each docking pose, the backbone movements are sampled over the putative interface, to both reduce computational time and capture realistic changes - this implies that the conformational change that occurs between binding partners is local, restricted to binding area. Whilst this is a possibility, it is not always the case since "off-site" conformational changes can be triggered by small interface changes and propagated to other sub-domains.

The reviewers are correct in stating that conformational changes in binding partners can be off-site, i.e., not limited in the binding interface domain. With induced fit replica exchange, our hypothesis with the putative interface has been to restrict the backbone motion only in a domain of interaction, specifically, an interface region where the protein partners can detect each other. While off-site conformational changes can be triggered by such binding-region interactions, unfortunately, allowing backbone flexibility for the entire protein structure proved to be detrimental and often resulted in unfolded protein structures (loss of secondary or tertiary structural elements, see p.7, line 159).

To clarify on the reviewer's comments in the manuscript, we added the following text in the Discussion section, paragraph 3:

"As ReplicaDock 2.0 is restricted to backbone sampling at putative interfaces, it fails to accommodate off-site conformational changes, for e.g., co-evolutionary residues triggering off-site domain motions or maintaining a fold in the tertiary protein structure."

We also include the clarification in Results section:
"To capture realistic backbone conformations, we therefore restrict backbone moves to an 8 Å local interface. Unfortunately, this selection precludes longer range, off-site conformational changes."

2. It is then discussed that expanding the selection to 8A increased the [performance] across all targets during local docking. Later, authors discuss that the induced fit can recover a high fraction of native contacts but the backbone is not observed to be closer to the bound conformations. For this analysis the structures generated in the low-resolution stages were used; it is unclear if the different criteria for expansion were also used here (during the low resolution stages) and how/if it would capture backbone conformations closer to the bound form.

We thank the reviewer for highlighting the lack of clarity here. To determine the optimal setting for backbone moves with the tests, we used the full protocol (i.e., low resolution + high resolution refinement) and Figure 2.B highlights the *Full Protocol average N5 scores* for the different cases. We added the following line in the text to reflect that (Results, *Flexible local docking with ReplicaDock 2.0 samples deeper energy funnels*):

"To evaluate the extent of flexibility that can be incorporated while docking for optimum performance, we tested ReplicaDock 2.0 protocol (low resolution sampling with high resolution refinement) with different selections of residues for backbone sampling."

With induced-fit, we observed that the push towards capturing native-like backbone conformation is limited, although there was a considerable increase in recovering native-like contacts (~0.2 points increase in fraction of native-like contacts). For the RMSD estimation, the heavy atom RMSDs of the receptor and ligand chains were measured (as shown in Fig. 3.B,E) after the full-protocol. No, alternative mobile residue sets (such as loops, or interface residues) were not used for the benchmarking task after we concluded that 8 Å interface is optimum. Since the high-resolution stage usually refines the structures by under 0.5 Å and we are estimating heavy-atom RMSDs in our backbone RMSD measurement, evaluating the RMSDs after low-resolution would not result in any difference in our conclusions.

3. The creation and optimization of the motif updated dock score is in line with the objective of testing the performance of the method. As stated in figure S6 - "for each of the 11 protein-protein complexes."; for completion, authors could include what were the complexes used for optimisation.

We now include information about the 11 protein-protein complexes in the supplementary in Figure S6:

Difficult targets	Medium targets	Rigid targets
1FQ1<br>3FN1<br>3H11	1GRN<br>1IJK<br>1LFD<br>3DAW<br>4FZA	1AY7<br>1MAH<br>2PCC<br>2SNI

4. The accuracy of the optimised MUDS function is tested on 10 protein targets and it is shown to out-perform ClusPro, however the authors do state the high computational cost of ReplicaDock 2.0. It should also be included what is the total (CPU) time to run the prediction of one target, using backbone conformational sampling.

The compute time for ReplicaDock 2.0 with backbone sampling is 8-72 hrs depending on the number of residues in the protein complex. We now include these numbers in the main text (Results, *Benchmark evaluation demonstrates improved performance*

over conformer-selection methods) with a Supplementary Figure 17 to demonstrate the scaling of ReplicaDock 2.0 and RosettaDock 4.0 with the residue size, for our benchmark targets. The following text is now included in the manuscript:

"Finally, we also compared the run time of ReplicaDock 2.0 with RosettaDock 4.0 for local docking across the benchmark targets. For all benchmark targets, we could generate the ReplicaDock 2.0 trajectories on our current hardware (24 processors) in a compute time of 8-72 CPU-hrs. The scaling of the ReplicaDock 2.0 and RosettaDock 4.0 protocols with the number of residues in the complex, N_{res}, is illustrated in Supplementary Fig. S17"

5. In the comparison with RosettaDock 4.0 it is shown that ReplicaDock 2.0 performs better, as expected, for moderately-flexible and (even if modestly) to the ones with higher conformational changes. In the supplementary material the individual metrics for each complex are shown, however they could be sorted by difficulty, grouping the easy/intermediate/hard and complex side, this could also reveal any inherent trends such as: how is the performance related to the complex size? A minor comment that could improve readability is that authors state that some of the complexes are "rigid", indeed in BM5 these are named as "rigid-body" however, there is, even if, a very small (>1A) conformational change for all cases, it could be clarified in the text or renamed.

We have changed Table S2 to include i-$RMSD_{unbound-bound}$ (Å).
The Supplementary Figures are in the order of difficulty, for eg:

- Figure S10: Low Resolution Docking performance for Rigid targets
- Figure S11: Low Resolution Docking performance for Medium targets
- Figure S12: Low Resolution Docking performance for Difficult targets

and so on for the High-resolution performance (Figs. S13 - S15). Within each section however, we have sorted the targets alphabetically for the ease of the reader. Alternately, we could sort by an evaluation metric like iRMSD, but that might be more difficult to find.

The reviewer is correct that rigid targets exhibit conformational changes, although under 1.2 Å.We changed the text to highlight the same (Methods: *Benchmarking, evaluation and success metrics*).
"Dockground Benchmark Set classifies protein targets as rigid ($RMSD_{unbound-bound}$ < 1.2 Å), medium (1.2 A < $RMSD_{unbound-bound}$ < 2.2 Å) and difficult targets ($RMSD_{unbound-bound}$ < 2.2 Å), depending upon the conformational change between unbound and bound structures."

6. The application of the directed induced fit mechanism yields very interesting results and seems to point that the protocol could be further improving by adding information in the form of "interface patches" - could these be used in combination with the default protocol, adding perhaps weights to the "biased" interface?

A. Providing information about interface patches:

Yes, the current protocol has accessibility to bias the backbone sampling towards more mobile regions. We have included the XML scripts for the directed induced fit in the `demos/public/replicadock2` directory with the Rosetta package *(Supplementary, Scripts and Tutorials).* To briefly elaborate, users can also explicitly input the residues that they want to be flexible during docking and the ReplicaDock2.0 protocol will perform backbone motion on only those residues.

We elaborate this in the Supplementary Information with the following text:
"The scripts for interface residue test selections, global docking, local docking and directed-induced fit are deposited in the `InterfaceTest`, `GlobalDock`, `LocalDock` and `directed_localdock` directories respectively. The global docking and local docking scripts would allow the users to reproduce our results or employ them for other docking systems. The directed induced-fit scripts would allow the users to bias sampling towards user-defined mobile residues."

B. Adding biased score weights:

We can provide a weighted bias to the bound-like interface in the scorefunction and it would improve the performance. However, it would not be blind docking, since the interface residues would already be identified. Instead, if the user has information about mobile residues, either by experiments (cross-linking experiments, binding studies, etc) or alternative computational metric (e.g., pLDDT, PAE, Root mean square fluctuation, etc), they could feed mobile residues identified externally into the protocol.

We now discuss this aspect of utilizing directed induced fit in the Discussion section:
"Thus, for blind targets, if we could identify potentially flexible residues from homologous structures or from AlphaFold's confidence metrics, such as predicted LDDT Cα score (pLDDT) or predicted TM Score (pTM), ReplicaDock 2.0 could be guided to allow targeted flexibility."

7. ReplicaDock 2.0 is a valuable addition to the community, going in an alternate route from the common conformer-selection showing that an induced-fit approach can result in near-native molecular recognition in flexible targets. The unfortunate bottleneck is the computational cost associated with the protocol, the outlook on this limitation as well as potential optimizations could be briefly discussed.

We thank the reviewers for their kind note and appreciation. We agree that the high computational cost is an unfortunate bottleneck, but it is still faster than more intensive methods such as MD or conformer selection methods such as RosettaDock 4.0. Backbone sampling involves a relatively larger sample space and so the approach is computationally expensive for larger targets, unless the appropriate regions of flexibility are pre-determined.

With our current hardware (with 24 processors), ReplicaDock 2.0 can yield local docking results within 8-72 hrs (depending on protein residue size), which is relatively faster (30-60% faster than RosettaDock 4.0) for large-scale protein docking studies capturing conformational changes (see Results, *Benchmark evaluation demonstrates improved performance over conformer-selection methods, highlighted above in comment 4*).

We have further included the following text in the Discussions section to acknowledge this limitation and provide alternative routes of optimization.

“Despite the improvement in docking performance, ReplicaDock 2.0 brings limited computational speed-up. Currently, ReplicaDock 2.0 generates local docking structures in 30-60% less time than RosettaDock 4.0. Yet, the high compute time is a caveat of the protocol, particularly for larger complexes, or complexes with higher interacting residues. As opposed to embarrassingly parallel approaches that can utilize higher compute power for a similar time-frame, ReplicaDock 2.0 requires 8-72 hrs. on 24 processors for a docking simulation. Although this is computational efficient over conformer-selection methods, such as RosettaDock 4.0 or MD simulations, by increasing sampling trajectories and utilizing multiple processors, we could improve run times without compromising on backbone sampling.”

#Reviewer 2

Summary: The ReplicaDock 2.0 protein-protein docking method introduced in this paper is aimed at the modeling of conformational changes induced by the binding partner (induced fit). In a previous effort, the Gray lab has introduced RosettaDock4.0 that addressed conformational flexibility as conformer ensembles. Here, the focus is put on binding-induced conformational changes. ReplicaDock 2.0 builds on replica-exchange MC-based (REMC) rigid-docking protocol (ReplicaDock, developed by Zhang et al.). The authors used an improved low-resolution scoring function (MUDS: Motif Dock Score adapted to include backbone movements) and introduced backbone sampling into REMC (using backrub moves). The protocol is assessed in the following way: first, rigid global docking was performed on a set of 10 protein targets starting from random orientations of the protein partners. The new low-resolution scoring function was shown to improve the sampling to identify local binding patches. Overall however, the improvements compared to RosettaDock4.0 in global docking are not very dramatic. It seems to improve fnat in global docking in a few challenging cases with medium-to-highly flexible backbones. It is computation intensive (e.g., 20x cluspro). Importantly, however, comparison of RosettaDock4.0 to ReplicaDock2.0 allows us to assess the contribution of conformer selection vs induced fit. Second, flexible local docking on a set of 12 complexes showed deeper sampling of energy funnels. The flexibility of the receptor backbone was optimized (use of an 8 Å interface patch was found to be optimal). However, despite generating lower energy structures, the improvement towards the native structure seems marginal. Lastly, for a large benchmark set of 88 complexes, the protocol improved predictions compared to RosettaDock4.0, for rigid, medium as well as flexible cases. However, improvement is still marginal, and for highly flexible complexes, sampling is not sufficient. This was improved with targeted sampling, when the flexible region (e.g. loop) is known. Given the marginal improvement of the blind protocol, contrasted to the significant improvement once the flexible regions are known, it is clear that one needs to investigate methods to identify these regions. Such possible directions are mentioned in the discussion section. Overall, this study presents an important step forward in the challenging path of flexible docking. Methods and results are presented clearly and in detail. I have only a few questions, and comments that could be addressed to improve the manuscript.

Major suggestion:

1. One topic that could be discussed more is the marginal improvement of high-resolution refinement. Is it indeed necessary, after introducing MDS (and MUDS)?

   The high-resolution, all-atomistic refinement is necessary not just for side-chain packing, but also to resolve any potential side-chain clashes or penalize orientations that MUDS failed to penalize. Further, it can result in smaller motions and side-chain rearrangements both throughout the structure as well as on the interface, resulting in a protein complex with a tighter fit. Moreover, with all-atomistic, high-resolution refinement, we ensure that the structures are the lowest possible thermodynamic state. This is important if the docking models would be employed for downstream

uses, such as protein design or re-engineer interfaces for altered specificity and binding.

As evidence of the importance of the high-resolution stage, we point the reviewers towards the Supplementary Figures S10-S12 (particularly, rigid targets 1MAH, 1MLC and 2BTF, medium targets 1FC2, 1IJW, 1MQ8, 2HRK, and difficult targets 1FQ1, 1PXV, 3F1P), where we have colored the low-resolution candidate structures with their final high-resolution CAPRI quality obtained post refinement. We include this in the Result section:

"Supplementary Figure S10-12 represent the low-resolution candidate structures colored with their final high-resolution CAPRI quality to show the improvement due to refinement. In multiple cases (e.g., medium targets 1MQS and 2HRK), the high-resolution stage penalizes poor, false-positive structures and refines near-native structures to improve their quality, showing that best results are achieved by combining the MUDS T-REMC stage with all-atom refinement."

Additionally we clarify the purpose of the refinement in methods and results. New text in the methods:
"The all-atom, high-resolution refinement resolves any side-chain clashes and penalizes false-positive orientations that the low-resolution score function failed to penalize. This ensures that our output structures are at the lowest possible energetic state achievable for the attained conformations"

New text in Results, *Induced-fit recapitulates native contacts but fails to push backbone sampling towards bound conformations*:
"With low-resolution sampling, ReplicaDock 2.0 explores larger conformational space in a rapid fashion and avoids entrapment in local minima. However, the structures generated are limited with their accuracy and often require all-atom refinement to penalize side-chain clashes or spurious interfaces and yield realistic structures. The all-atom refinement can further lead to smaller motions and side-chain rearrangements that can result in compact binding between protein partners."

Other suggestions:

2. The protocol does not include any "prepacking" step, in which internal clashes of the receptor and ligand are removed before the start of the docking procedure. If backbone changes lead to changes in internal energy, then a docking trajectory could in principle result in different local relaxations that affect the evaluation score. Could the authors comment on this?

Thanks for noticing that we omitted this detail. In preparing the targets, we indeed repacked side chains of each unbound structure using Rosetta FastRelax with relax:bb_move false flag. The relaxation protocol includes side-chain prepacking that

resolves internal clashes, so we do not need to explicitly perform prepacking prior to docking.
To clarify this in the manuscript, we have further included the following text in the manuscript, Methods: Generation of Initial conformations:

To generate this starting pose, we randomize the initial orientation of the protein partners (unbound monomers prepacked with Rosetta FastRelax) with the Rosetta option `randomize1`, `randomize2` and `spin` (details in the sample XML script).

We also include the following text in Supplementary methods, *Benchmark Sets*:

“Next, we used Rosetta FastRelax on each unbound protein monomer with `relax:bb_move false` to perform side-chain packing and resolve internal clashes."

“Note that as opposed to RosettaDock, we do not need to explicitly perform a pre-packing step as backbones are sampled in conjunction with docking. For conformer selection, since ensembles are pre-generated, the pre-packing step informs the docking protocol of the inherently different energies of the conformers in the ensemble. In ReplicaDock2.0, the changes in backbones are evaluated with the energy function at each step and we assess the candidate structures based on their interface scores.”

3. For global docking, a constant number of decoy trajectories are sampled (n=24k). Does this affect performance for larger complexes? Could scaling this based on the receptor surface improve efficiency and performance?

Yes, the MC trial step-size can affect performance for larger complexes. For proteins with a larger residue size (and bigger interface), higher number of trials would lead to better sampling. In our study, global docking is rigid-backbone so we could run itlonger, with more frequent exchanges and search the entire protein surface without distorting the protein structure.

Scaling it based on receptor surface or number of residues would improve performance. However, considering the compute time utilized for the global docking approach, using a slightly less accurate but considerably faster FFT-based rigid docking approach might be a better alternative to obtain a starting point for local docking runs with backbone motions - a task that we have benchmarked our protocol for, and have obtained better performance than other methods. We have also included a new scaling plot in Supplementary Figure 17, that shows the scaling of the local docking protocol (with backbone sampling) for the protein targets to generate one decoy with respect to the residue size. This plot further illustrates the point made by the reviewers about residue size and number of interface residues affecting performance and run-times.

We have included the following text in Discussion section to elaborate on the computational efficiency of the protocol and potential routes for optimization:

“Despite the improvement in docking performance, ReplicaDock 2.0 brings limited computational speed-up. Currently, ReplicaDock 2.0 generates local docking structures in 30-60% less time than RosettaDock 4.0. Yet, the high compute time is a caveat of the protocol, particularly for larger complexes, or complexes with higher interacting residues. As opposed to embarrassingly parallel approaches that can utilize higher compute power for a similar time-frame, ReplicaDock 2.0 requires 8-72 hours on 24 processors for a docking simulation. Although this is computational efficient over conformer-selection methods, such as RosettaDock 4.0 or MD simulations, by increasing sampling trajectories and utilizing multiple processors, we could improve run times without compromising on backbone sampling.”

4. Also related to sampling (Page 14): for generating starting conformations for local refinement, it seems that the perturbation involves only translation on the axis that connects the two centers of mass (moving 15A away), but no translation in the other two dimensions. Please clarify that this is the case and discuss whether this indeeds warrants random local sampling.

For global docking, the two proteins are randomized with respect to each other, resulting in completely blind global docking. For local docking, since we seek simply to remove memory of the native state, we move the two unbound protein structures along their center of mass up to 15 Å away and rotating the smaller partner by 45° to distort the binding interface. Each trajectory also adds a random perturbation of 1 Å and 1° to provide slightly different starting states.

We have clarified this in the text in the Methods section, *Generation of initial conformations.*
“Then, we move the unbound monomers 15 Å away from each other with a 45° rotation to the ligand (smaller monomer) with respect to the receptor. This serves as the input structure to the ReplicaDock2.0 protocol. For each trajectory, a Gaussian random 1 Å and 1° perturbation provides slightly different starting states. We have observed that higher temperature replicas often result in much broader exploration of the protein surface.”

We also clarify the docking scope in the text in the Methods section:

“The docking challenge can be categorized dependent on two scopes, namely, (1) Global docking, where there is no prior knowledge about protein binding, and (2) Local docking, where we have limited information about the binding regions. Global docking challenges are blind protein docking challenges involving prediction of the potential binding sites or orientations. After identifying potential binding regions, local docking aims to narrow down the scope to a localized region of protein to predict the conformations of complexes with better confidence.”

5. Clustering: the results are presented without clustering, leading to cases where we have either N5=5 or N5=0 (at least in the low resolution step). In cases of N5=0, would clustering help?

The reviewer is correct in suggesting that clustering could assist in identifying a representative set of top N decoys. However, for almost all cases with N5=0 (see Supplementary Figures S13-S15, e.g., difficult targets 1EER, 1F6M, 1FAK), the backbone sampling is inadequate in capturing native-like conformations i.e., no decoys under 5 Å i-RMSD (in high resolution) are sampled. Clustering thus would not be helpful in these cases as the limitation is backbone sampling not the selection of top N decoys.

6. From the figure, it is not clear how much the high-resolution step improves the results. Can the authors discuss this more? In addition, could maybe bb minimization at the high-res step help? Or would it just increase runtime?

To highlight the important of the high-resolution stage, we point the reviewers towards the Supplementary Figures S10-S12 (particularly, rigid targets 1MAH, 1MLC and 2BTF, medium targets 1FC2, 1IJW, 1MQ8, 2HRK, and difficult targets 1FQ1, 1PXV, 3F1P), where we have colored the low-resolution candidate structures with their final high-resolution CAPRI quality obtained post refinement. In multiple cases, such as the ones listed above, high-resolution penalizes false-positive minima and refines near-native structures (including some decoys over 5 Å RMSD) to bring them to a CAPRI acceptable quality or above.

We include this in the Result section:

“Supplementary Figure S10-12 represent the low-resolution candidate structures colored with their final high-resolution CAPRI quality to show the improvement due to refinement. In multiple cases (e.g., medium targets 1MQS and 2HRK), the high-resolution stage penalizes poor, false-positive structures and refines near-native structures to improve their quality, showing that best results are achieved by combining the MUDS T-REMC stage with all-atom refinement.”

The high-resolution stage performs smaller rigid body motions accompanied by interface sidechain packing and energy minimization in torsion angle space (ϕ, ψ and χi).

We have elaborated on this part in detail in the Results by editing the following text: “After generation of candidate docking poses in the low-resolution stage, the high-resolution stage performs an all-atom refinement which employs finer rigid-body motions (random rigid-body perturbations in a Gaussian distribution of 0.1 Å and 3°) with side-chain rearrangements followed by energy minimization in the torsional space. This stage does not explicitly move the backbone of the docked proteins but

resolves any side-chain clashes and forms a compact, low-energy, high-resolution interface"

Technical details:

7. References: Move reference #2 from line 9 to line 13 (is about flexible docking, less about PPI networks). The supplementary material is missing the bibliography.

We have made the changes and included the Supplementary bibliography, Page 52 in Supplement.

8. Figure 3B, E: include as "x" the starting RMSD of the receptor and ligand – will allow to assess whether we move into the right direction.

We have now included bars indicating the RMSD of the unbound partner in Figure 3.B and E.

9. Figure 4A: define the three numbers included at the top of the figure explicitly (better, equally, and worse-modeled complexes).

We thank the reviewers for pointing this out. We have updated the caption to included Better, Equal and Worse for comparison.

10. Figure 4B: the density plots of the dark green and light green lines (ReplicaDock2 high res and low res) don't cover the same area: how come?

    The plot in Figure 4B reflects the <N5> metric probability densities for both the high resolution and low-resolution stages of ReplicaDock and RosettaDock. N5 denotes the number of structures in top 5 scoring structures being near-native. However, the criteria to determine whether a structure is near native is different for both high- and low-resolution stages (as described in Methods, *Benchmarking, evaluation and success metrics*). For low resolution, this criterion only requires the sampled structures to be under 5 Å Cα-RMSD. On the other hand, for high-resolution structures, we need a CAPRI-acceptable quality or above. Since the methodologies are different, the area under curve is expected to be different. However, with the density curves, our aim is to highlight an overarching trend in the performance.

    We clarify this in the main text, Results section as follows:
    "With the probability density curves, since the criterion to determine a near-native structure is different for high-resolution and low-resolution stages, the area under curve (AUC) differs. However, it can capture some overarching trends in performance: ReplicaDock 2.0 shifts the curve towards higher <N5>, particularly for moderately-flexible targets."

11. In Table S2, highlight the pdbs that are included in the n=12 dataset in Fig 2B and n=11 in Figure S6.

The protein targets used for score function optimization are now listed in Figure S6. We have additionally included the PDBs for interface optimization in the Figure S4.

Difficult targets	Medium targets	Rigid targets
1FQ1<br>1JK9<br>3F1P<br>3FN1	1GRN<br>1IJK<br>3DAW<br>4FZA	1AY7<br>1MAH<br>2PCC<br>2SNI

12. Figure S3: Please define the label "Gray". I assume it refers to ReplicaDock2 reported here.

We have updated the caption label to denote "gray-tan" as the bound structure.

13. Figure S6: label (a) and (b), or indicate "left" and "right"

We have updated it to left and right respectively.

Reviewer #3:

The authors tackle an essential problem of protein docking, which can provide crucial insights into subsequent protein functions and details on protein-protein interaction.

The described method, ReplicaDock 2.0, is a method that combines induced fit (IF) binding mechanisms with replica exchange, extending the existing rigid-docking approach with backbone motions. This allows for a more precise estimation of flexible backbone target positioning. Replica Dock 2.0 reports an impressive ~60% success rate on the targets with moderate flexibility.
Even though the paper describes a combination of the existing methods – temperature Replica-exchange Monte Carlo and Rosetta Backrub during low- and high-resolution steps of the algorithm, the overall protocol is novel and demonstrated an advantage in the prediction of protein complexes for targets with medium and- high backbone flexibility. Another notable contribution of the authors is the development of Motif Updated Dock Score (MUDS) that extends the existing Motif Dock (MDS) by incorporating knowledge-based backbone torsion statistics and Van der Waals interaction. The authors conducted a thorough comparison of the ReplicaDock 2.0 with the existing docking methods, including deep learning-based RoseTTAFold and AlphaFold-Multimer, and defined its area of applicability. Benchmark datasets used, such as Docking Benchmark DB5.5 and recent CAPRI challenge rounds, are the community's golden standard. Deliverables are part of the Rosetta community tool, which facilitates sharing of the tool and its dissemination in the community.

1. There is a question regarding the docking benchmark dataset. Authors report results for ReplicaDock 2.0 and RosettaDock 4.0 on the same dataset – Docking Benchmark 5.0. The number of easy, medium, and difficult targets differ in Supplementary Table 1. It seems like three easy targets were moved to medium and difficult categories, but the rationale behind this is not clear.

Our presentation of the numbers in ST1 was unclear. In Docking Benchmark 5, there are 151 rigid (easy) targets, 45 medium-difficulty targets and 34 difficult targets. Out of these RosettaDock tested on 13 rigid, 43 medium and 32 difficult. Since our main aim was to check on more flexible targets, we focused on all medium (45) and difficult (34) targets and just 10 of rigid targets. Out of the 45 medium targets, protein target 3R9A was excluded as obtaining ensembles for 3R9A in reasonable time was infeasible.

So, we haven't moved easy targets to medium or rigid, but instead included relatively more flexible than rigid targets. Coincidently, the number of targets that we reduced from rigid matched the number we increased in medium and high cases. We have included a note in the caption to indicate that the same.

"The ReplicaDock 2.0 and RosettaDock 4.0 test sets differ slightly because we omitted some easy targets and we added flexible targets that had been too large for the prior ensemble methods."

2. Another question is regarding the computational time. Authors give a comparison in CPU hours with ClusPro, but not with the RosettaDock 4.0, another method extensively used in the paper as a baseline, or molecular dynamics, that should give more precise results but is exceedingly slow. In my opinion, listing estimates for CPU hours in global and local scope for those methods would help readers select an optimal tool for the specified level of complexity.

We now include a plot in the Supplementary (Supplementary Figure 17) that shows the observed scale-up of ReplicaDock 2.0 with RosettaDock 4.0 (with and without ensembles). We introduce these data with new text in Results, *Benchmark evaluation demonstrates improved performance over conformer-selection methods,* and Discussion sections respectively:

"Finally, we also compared the run time of ReplicaDock 2.0 with RosettaDock 4.0 for local docking across the benchmark targets. For all benchmark targets, we could generate the ReplicaDock 2.0 trajectories on our current hardware (24 processors) in a compute time of 8-72 CPU-hrs. The scaling of the ReplicaDock 2.0 and RosettaDock 4.0 protocols with the number of residues in the complex, N_{res}, is illustrated in Supplementary Fig. S17"

"Despite the improvement in docking performance, ReplicaDock 2.0 brings limited computational speed-up. Currently, ReplicaDock 2.0 generates local docking structures in 30-60% less time than RosettaDock 4.0. Yet, the high compute time is a caveat of the protocol, particularly for larger complexes, or complexes with higher interacting residues. As opposed to embarrassingly parallel approaches that can utilize higher compute power for a similar time-frame, ReplicaDock 2.0 requires 8-72 hrs on 24 processors for a docking simulation. Although this is computational efficient over conformer-selection methods, such as RosettaDock 4.0 or MD simulations, by increasing sampling trajectories and utilizing multiple processors, we could improve run times without compromising on backbone sampling."

3. Lastly, I would have liked to see the authors providing a better rationale of why the approach is innovative and provides substantial (not gradual) improvement to warrant a publication in PLOS CB. If I were to play devil's advocate, it is just another docking method with no drastic improvement over other methods and is not really using a radically new method. So, what warrants it to be published here?

Our method is innovative and important because:

1. Our work represents the first instance of a replica exchange Monte Carlo (REMC) docking routine that exhibits backbone motions. Building on prior work including REMC and MDS, we combine these advances for the first time with on-the-fly backbone motions for docking. The docking conformational space increases exponentially with backbone flexibility, and so it is challenging to move the backbones while generating realistic structures within a feasible compute time. We

overcame this challenge by using temperature replica exchange with a fast-scoring low-resolution score function and interface-specific backbone moves. (Line 52)

2. Protein-protein docking with backbone flexibility via induced fit for moderate to large scale motions has not yet been reported. Prior studies involving induced-fit motions have been limited to side-chain packing or small-scale, limited backbone motions. Further, in case where relatively large moves were performed, such as loop motions in Wang et al. (2007), the docking objective was already fulfilled and loops were remodeled over docked structures. ReplicaDock2 mimics induced fit by moving the backbone in conjunction with docking to—for the first time—consistently reach motions beyond 1 Å in the protein backbone. (New sentences added to discussion line 308)
3. For scientific reproducibility, it is critical to publish benchmark results on broad and challenging tasks. There are no other groups reporting performance on this broad set of medium and difficult (i.e., flexible) targets.
4. On this very hard problem, our improvement is significant. The improvement for moderate targets is from 49% (in RosettaDock 4.0) to 61% (in ReplicaDock 2). No other docking research groups have tested on all the moderate targets or shown success rates over 50% in this class of targets. (Discussion, line 288)
5. With directed induced fit, we demonstrate with examples that ReplicaDock2's backbone moves can reach the native-like state if the focus is on the mobile residues. This observation will guide future docking approaches based on physical modeling. (Discussion, lines 296-298)

We thank the reviewers for the opportunity to highlight these innovations of our work!
